# Cross national pharmacovigilance analysis of drug-induced delirium: a comparative study using the US FAERS, Japanese JADER, and canadian CVAR datasets

**DOI:** 10.3389/fphar.2026.1878501

**Published:** 2026-09-03

**Authors:** Jinghua Jiao, Shunming Liu, Ying Fang, Xin Song, Xingpeng Zhang, Jian Wang

**Affiliations:** 1 Department of Anesthesiology, The Affiliated Panyu Central Hospital, Guangzhou Medical University, Guangzhou, China; 2 Department of Ophthalmology, Guangdong Eye Institute, Guangdong Provincial People’s Hospital (Guangdong Academy of Medical Sciences), Southern Medical University, Guangzhou, China; 3 Department of Orthopedics, Shanghai Pudong New Area People’s Hospital, Shanghai, China

**Keywords:** disproportionality analysis, older adults, pharmacovigilance, spontaneous reporting systems, time-to-onset

## Abstract

**Background:**

Drug-induced delirium is a clinically important and potentially preventable adverse event, especially in older adults, yet large-scale cross-national pharmacovigilance evidence remains limited.

**Methods:**

We conducted a retrospective analysis using three major spontaneous reporting systems: the FDA Adverse Event Reporting System (FAERS, 2004–Q3 2025), the Japanese Adverse Drug Event Report (JADER, 2004–Q2 2025), and the Canada Vigilance Adverse Reaction database (CVAR, 2004–Q3 2025). Delirium cases were identified using five pre-specified Preferred Terms (PTs) selected from the narrow scope of the Standardised MedDRA Query (SMQ) Noninfectious encephalopathy/delirium. Disproportionality analyses were performed using reporting odds ratio, proportional reporting ratio, and empirical Bayes geometric mean, with signal strength defined by lower confidence bounds. Time-to-onset (TTO) was summarized using medians, IQRs, and early-onset proportions.

**Results:**

A total of 263,327 reports were identified (FAERS: 243,572; JADER: 7,512; CVAR: 12,243). Delirium was more frequently reported in females (49%–57%) and individuals aged ≥60 years (55%–70%). Strong and consistent signals were observed for nervous system drugs, particularly psycholeptics, analgesics, and psychoanaleptics. Duloxetine, lamotrigine, pregabalin, quetiapine fumarate, and zolpidem demonstrated consistent signals across databases. Additional signals involved antipsychotics, benzodiazepines, opioids, and selected anti-infective agents. Time-to-onset analysis demonstrated early-onset clustering, suggesting an acute temporal pattern.

**Conclusion:**

Cross-database consistency in drug class signals and early-onset temporal patterns highlights the need for medication review and risk mitigation in high-risk populations.

## Introduction

Delirium is an acute and fluctuating neuropsychiatric syndrome characterized by disturbances in attention, awareness, and cognition (Adamis et al., 2007; Wilson et al., 2020). It is associated with increased morbidity (Ely et al., 2004), prolonged hospitalization (Ely et al., 2004), long-term cognitive decline (Pandharipande et al., 2013), and substantial healthcare costs (Leslie et al., 2008). Although delirium is frequently observed among hospitalized older adults and critically ill patients (Inouye et al., 2014), drug-induced delirium represents a potentially preventable and clinically significant subtype (Alagiakrishnan and Wiens, 2004).

A wide range of pharmacological agents have been associated with delirium, including anticholinergics, sedative-hypnotics, opioids, corticosteroids, and various cardiovascular and metabolic medications (Catic, 2011; Harrison et al., 2020; Marvanova, 2016; Smirnov et al., 2025). However, establishing causal associations remains challenging due to the multifactorial nature of delirium, polypharmacy in high-risk populations, and the underrepresentation of vulnerable groups in cohort study (Kurisu et al., 2020). Furthermore, pre-marketing clinical trials are often underpowered to detect rare or delayed neuropsychiatric adverse events (Berlin et al., 2008).

Post-marketing pharmacovigilance databases offer an opportunity to evaluate real-world adverse drug reactions across diverse populations. Large spontaneous reporting systems such as the U.S. FDA Adverse Event Reporting System (FAERS), the Japanese Adverse Drug Event Report (JADER) database, and the Canada Vigilance Adverse Reaction (CVAR) database enable large-scale signal detection through disproportionality analysis. These databases allow for the assessment of demographic distributions, temporal trends, and time-to-onset (TTO) patterns, thereby facilitating early identification of high-risk drugs and vulnerable populations.

Despite the clinical burden of drug-induced delirium, few studies have comprehensively integrated multiple international pharmacovigilance databases to systematically evaluate drug-associated delirium signals. Cross-database validation is particularly important to enhance signal robustness and minimize region-specific reporting bias.

Therefore, this study aimed to (1) characterize the demographic and temporal distribution of drug-induced delirium reports; (2) detect and prioritize potential drug safety signals using disproportionality analyses; (3) evaluate time-to-onset patterns of delirium across drug classes. By integrating three major pharmacovigilance databases, we sought to provide a robust real-world evidence framework to inform clinical risk stratification and pharmacovigilance strategies.

## Methods

### Study design and data sources

This retrospective pharmacovigilance study analyzed post-marketing adverse event reports from three databases including FAERS, JADER database, and CVAR Online Database, which are public pharmacovigilance databases that compile spontaneous reports of adverse events related to drugs, vaccines, and other medical products. Specifically, FAERS collects spontaneous reports of adverse events and medication errors submitted to the U.S. FDA. The JADER database provides post-marketing spontaneous adverse drug reaction reports submitted to the Pharmaceuticals and Medical Devices Agency (PMDA) in Japan since 2004, while CVAR contains voluntary reports of suspected adverse drug reactions in Canada. We collected data on drug-induced delirium from three pharmacovigilance databases: FAERS (2004–Q3 2025), CVAR (2004–Q3 2025), JADER (2004–Q2 2025). FAERS and CVAR are updated quarterly, whereas JADER follows a semi-annual release cycle; the most recent JADER release available at the time of analysis contained data through Q2 2025, resulting in a one-quarter discrepancy.

### Case identification and inclusion criteria

The identification of delirium cases was based on the Medical Dictionary for Regulatory Activities (MedDRA) version 28.1. Delirium cases were identified using five MedDRA Preferred Terms selected from the narrow scope of the Standardised MedDRA Query (SMQ) Noninfectious encephalopathy/delirium: Delirium, Confusional state, Disorientation, Encephalopathy, and Mental status changes (Yamada et al., 2025; Tang et al., 2025). Of the 44 PTs in the narrow scope, these five were selected as the most diagnostically specific terms representing primary delirium presentations. These five PTs were applied consistently across all three databases (FAERS, JADER, and CVAR). To maintain the rigor of the signal detection process, we restricted our analysis to suspect drugs (Primary Suspect [PS] for FAERS and JADER; Suspect for CVAR). Reports with missing age or sex data were retained in the analytic dataset; demographic percentages were calculated among reports with non-missing values.

### Variables and outcomes

Demographic variables extracted included age, sex, and age group classification (<60 and ≥60 years). Temporal variables included year of report. Drug-related information included generic and brand names, therapeutic classes, and suspected drug roles. Event characteristics included time-to-onset (TTO), defined as the interval between drug initiation and the onset of delirium.

### Statistical analysis

Duplicate reports within each database were identified and removed prior to analysis. For FAERS, duplicate reports were removed using the built-in deduplication method of the faers R package, following FDA-recommended within-source and across-source duplicate detection procedures. For JADER, cases in the PMDA cancel list were excluded, and each primaryid was verified to retain only the highest report_count (latest version). For CVAR, Health Canada data are released with version_no-based management; programmatic verification confirmed that each report_id was unique. All statistical analyses were performed using R (version 4.4.0) and Python (version 3.11). The distribution of delirium events was summarized by age group, sex, and year of report. Temporal trends were visualized to assess changes in reporting patterns over time. To align data structures across databases, CVAR drug names were standardized to active ingredients (prod_ai) using the canada-vigilance-med-norm pipeline (Chalabianloo et al., 2025), as CVAR does not provide a standardized prod_ai field unlike FAERS and JADER. ATC classification for all three databases was performed using the WHO ATC/DDD Index. Disproportionality analyses were conducted using four statistical measures: Reporting Odds Ratio (ROR), Proportional Reporting Ratio (PRR), Information Component (IC), and Empirical Bayes Geometric Mean (EBGM). All metrics were derived from standard two-by-two contingency tables for each drug–event pair. A drug–event pair was considered a positive signal only when all predefined criteria were simultaneously satisfied: ROR ≥ 2 with the lower bound of the 95% CI > 1 and ≥ 3 reports; PRR ≥ 2 with the lower bound of the 95% CI > 1 and ≥ 3 reports; and EBGM > 2 with the lower limit of the one-sided 95% credibility interval > 1. IC was additionally calculated, with positivity defined as IC025 > 0; however, IC results were evaluated descriptively and were not incorporated into the composite signal determination. This conservative multi-criteria framework was adopted to identify robust and statistically stable drug–event associations while reducing spurious signals related to reporting bias or sparse data.

For each measure, signal strength was classified as Strong (lower bound ≥ 5), Moderate (2 ≤ lower bound < 5), Weak (1 ≤ lower bound < 2), or Negative (lower bound < 1). Composite signal strength was assigned hierarchically: Strong required all three measures to be Strong; Moderate required EBGM to be Moderate with at least one other measure also Moderate; remaining pairs with EBGM lower bound ≥ 1 were classified as Weak; all others were Negative.

Reports with TTO < 0 or > 365 days were excluded to limit bias from implausible or delayed reports. Median TTO, interquartile range (IQR), and proportions of cases occurring within 7 and 14 days were calculated for each drug and ATC level. Temporal risk patterns were classified as strong acute (median ≤7 days, IQR ≤14 days, and ≥60% within 7 days), moderate (median ≤21 days or ≥30% within 7 days or ≥50% within 14 days), or weak/delayed (others). Analyses were restricted to FAERS and JADER due to unavailable timing data in Canada Vigilance. Categories with <5 reports were considered unstable, and those with <10 reports were interpreted cautiously.

Packages openEBGM and tidyverse supported signal detection, data processing, and visualization. Linear regression was applied for trend analysis (including overall trends, top 10 ATC Level 1 and Level 2 classifications, top 10 individual drugs). Subgroup analyses stratified data by age (<60 vs. ≥ 60 years) and sex to evaluate signal consistency.

This study adhered to the READUS-PV guidelines (Fusaroli et al., 2024a; Fusaroli et al., 2024b).

## Results

### Baseline characteristics and demographic distribution

**FIGURE 1:**
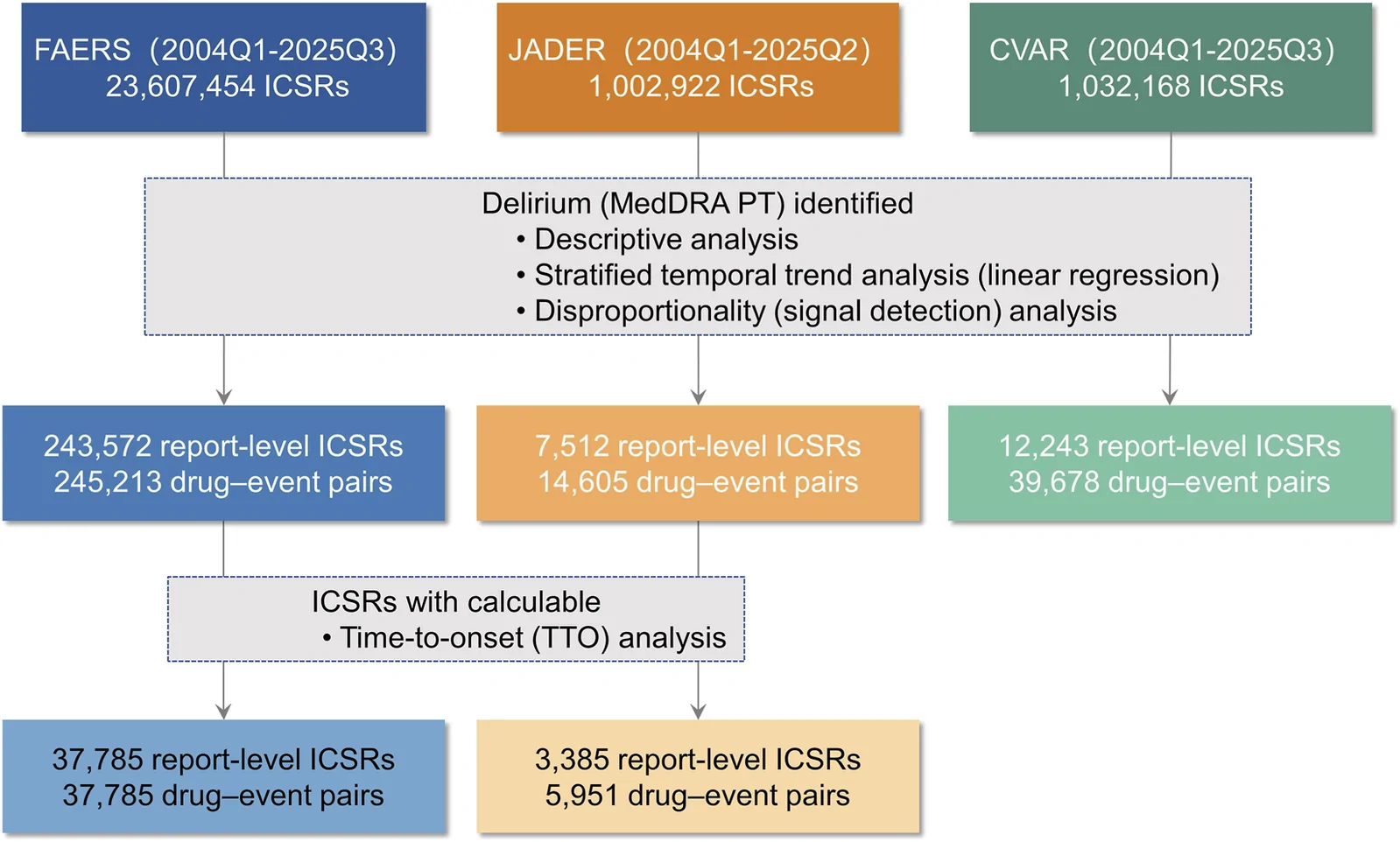
Study flowchart for identification of drug-associated delirium reports across FAERS, JADER, and Canada Vigilance databases.

A total of 263,327 individual case safety reports (ICSRs) of drug-associated delirium were identified during the study period, including 243,572 reports from FAERS, 7,512 from JADER, and 12,243 from the Canada Vigilance database (Figure 1). Across all regions, a consistent demographic pattern was observed. Female patients accounted for 55.5% in FAERS (n = 124,398), 48.8% in JADER (n = 3,460), and 57.2% in CVAR (n = 6,835). Delirium was predominantly reported in older adults (≥60 years), comprising 56.2% of cases in FAERS (n = 99,993), 70.0% in JADER (n = 4,996), and 55.2% in CVAR (n = 6,055) (Supplementary Figure S1).

### Trends of drug related–delirium

**FIGURE 2:**
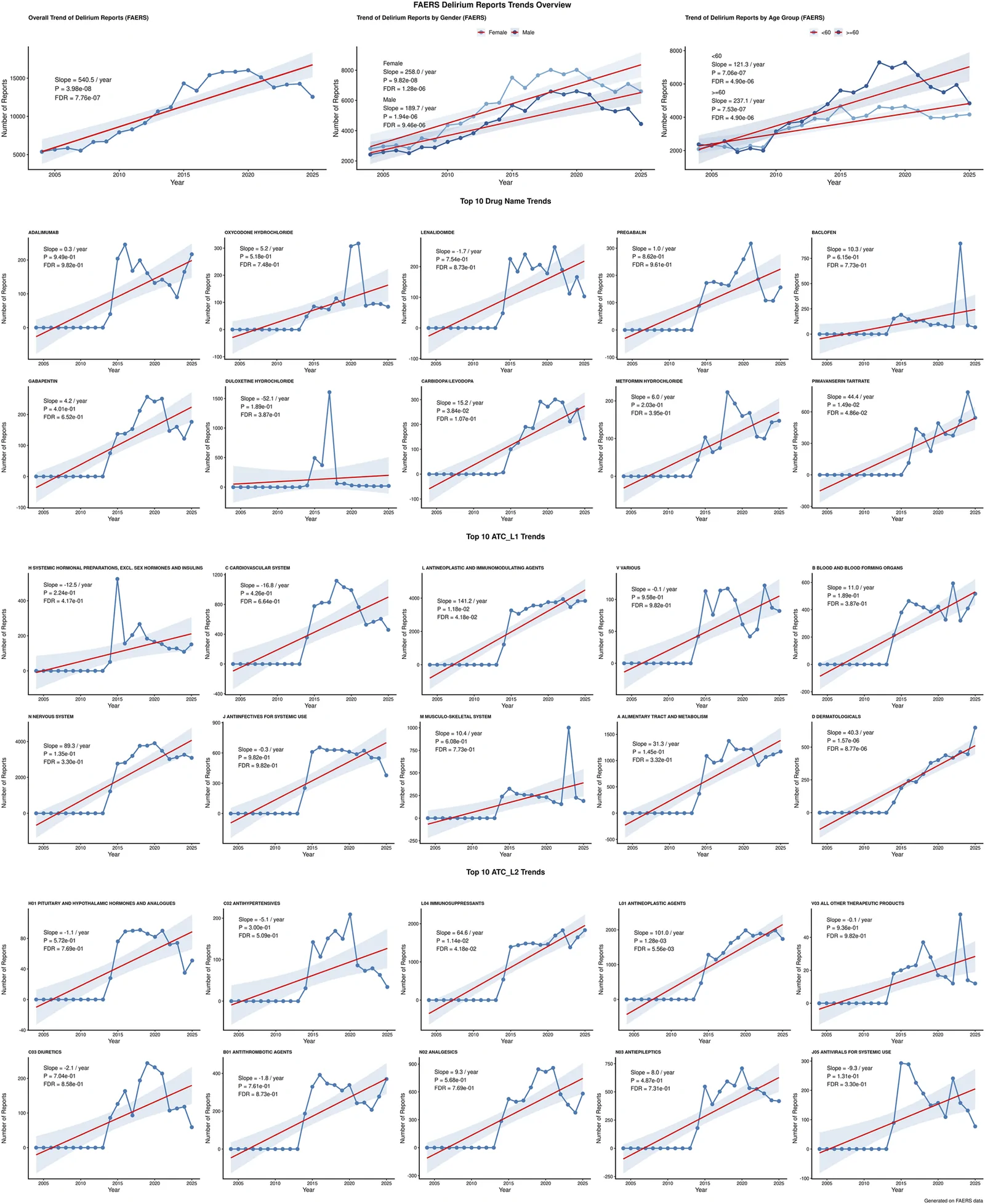
Linear regression trends of drug-associated delirium report counts in FAERS.

**FIGURE 3:**
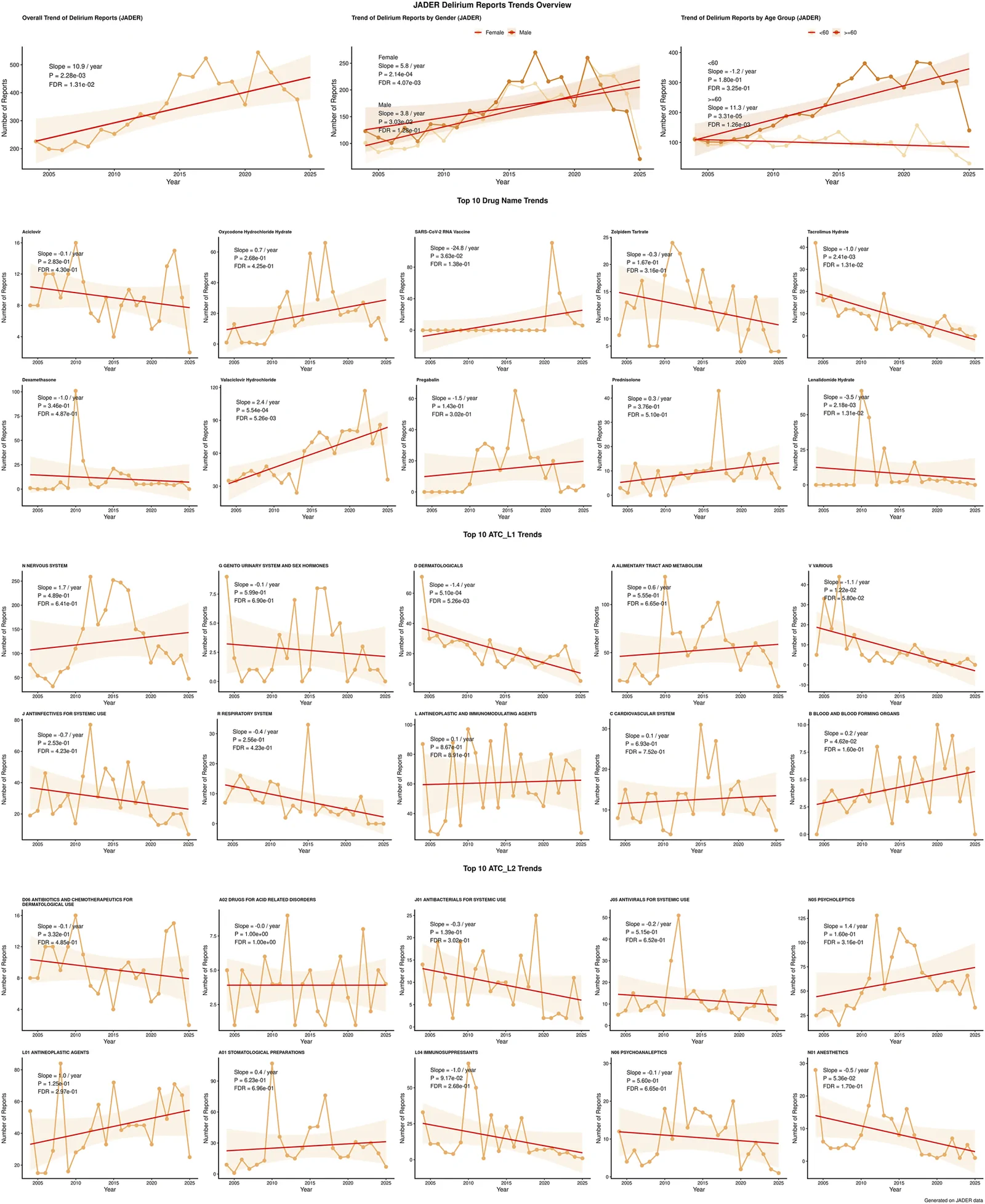
Linear regression trends of drug-associated delirium report counts in JADER.

**FIGURE 4:**
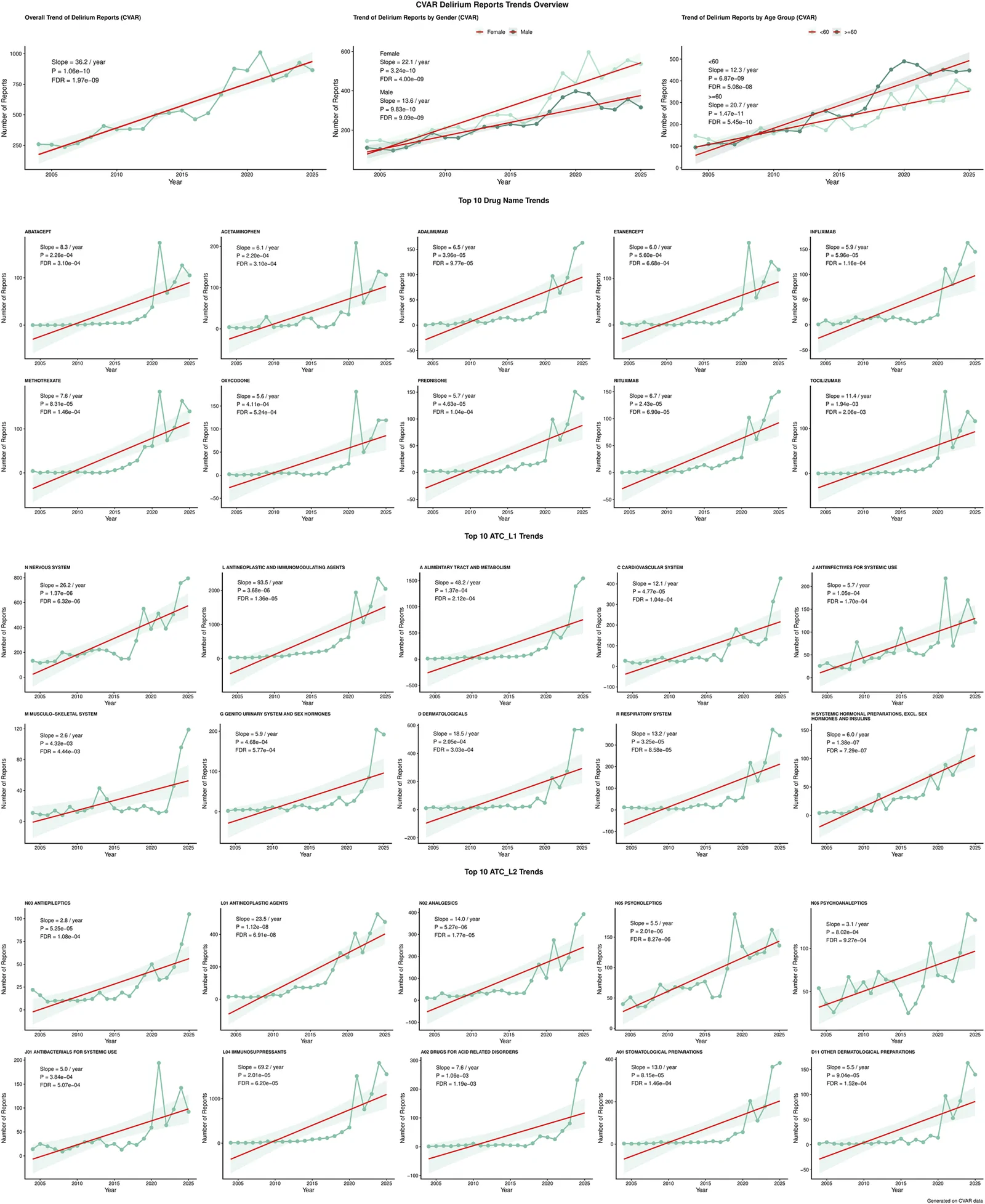
Linear regression trends of drug-associated delirium report counts in Canada Vigilance.

Drug related-delirium reports demonstrated significant upward trends between 2004 and 2025 (Supplementary Table S8). The increase was most pronounced in FAERS (annual slope 540.5 reports/year, p < 0.001) (Figure 2) and Canada Vigilance (36.2 reports/year, p < 0.001), while JADER showed a modest rise with statistical significance (10.9 reports/year, p = 0.002) (Figures 3, 4).

Age-stratified analyses revealed steeper annual increases among older adults (≥60 years) compared with younger individuals across all three databases. In FAERS, the slope for older adults was nearly double that of younger patients (237.1 vs. 121.3 reports/year, both p < 0.001). Similar patterns were observed in JADER (≥60 years: slope 11.3, p < 0.001; <60 years: slope −1.2, p = 0.18) and Canada Vigilance (≥60 years: slope 20.7, p < 0.001; <60 years: slope 12.3, p < 0.001).

In FAERS, while nervous system drugs and analgesics accounted for a substantial volume of reports, statistically significant upward trends were specifically observed in dermatologicals (p < 0.001) and antineoplastic agents (ATC L01, p = 0.001). At the drug level, pimavanserin tartrate (slope: 44.4, p = 0.015) and carbidopa/levodopa (p = 0.038) displayed significantly increasing trajectories (Figure 2).

JADER exhibited a relatively stable long-term increase, characterized by notable transient peaks in 2021–2022 primarily associated with SARS-CoV-2 RNA vaccines. Beyond these peaks, significant growth was also observed for specific agents such as valaciclovir (slope: 2.4, p < 0.001) (Figure 3).

In Canada Vigilance, a pronounced linear increase was observed, driven largely by immunosuppressants (ATC L04; slope 69.2, p < 0.001), antineoplastic agents, analgesics, psycholeptics, nervous system drugs, and anti-infectives. Acceleration was particularly evident after 2015 (Figure 4).

### Disproportionality analysis and signal detection

Disproportionality analyses identified high-priority signals predominantly among nervous system drugs (ATC Level 1), showing consistent positivity across FAERS, JADER, and Canada Vigilance. At ATC Level 2, psycholeptics, analgesics, and psychoanaleptics were positive in all three databases, whereas antiepileptics showed signals in FAERS only. Cross-database consistency (≥2 databases) was observed for five drugs—duloxetine hydrochloride, lamotrigine, pregabalin, quetiapine fumarate, and zolpidem tartrate (Supplementary Table S1).

Database-specific analyses revealed distinct patterns (Supplementary Figure S2). FAERS demonstrated the broadest signals driven by centrally acting drugs, including baclofen (ROR 12.00; EBGM_lower = 10.20), duloxetine hydrochloride (ROR 9.57; EBGM_lower = 8.35), pimavanserin tartrate (ROR 9.33; EBGM_lower = 8.15), lorazepam (ROR 6.41), tramadol (ROR 5.79), zolpidem tartrate (ROR 5.50), and quetiapine (ROR 5.33) (Supplementary Table S2).

In JADER, exceptionally strong signals were observed for anti-infectives and opioids, including valaciclovir hydrochloride (ROR 47.11), tapentadol hydrochloride (ROR 41.76), oxycodone hydrochloride hydrate (ROR 33.73), aciclovir (ROR 14.87), fentanyl (ROR 14.67), morphine hydrochloride hydrate (ROR 12.10), zolpidem tartrate (ROR 12.04), cefepime dihydrochloride hydrate (ROR 9.32), and donepezil hydrochloride (ROR 4.55) (Supplementary Table S3).

Canada Vigilance signals featured immunomodulatory/immunosuppressive agents and analgesics, including oxycodone (ROR 8.32), acetaminophen (ROR 7.65), leflunomide (ROR 3.28), prednisone (ROR 2.81), tocilizumab (ROR 2.60), and abatacept (ROR 2.38) (Supplementary Table S4).

### Time-to-onset analysis

In FAERS, most ATC Level 1 classes showed short median TTO values (Supplementary Tables S5, S6). VARIOUS exhibited the strongest acute profile (median 0 days, IQR 0–1; 89.2% ≤ 7 days), classified as strong, alongside antiinfectives for systemic use (median 3 days, IQR 0–10; 69.1% ≤ 7 days). Most other classes—including nervous system drugs (median 2 days), alimentary tract and metabolism drugs (median 2 days), and musculo-skeletal system drugs (median 2 days)—were categorized as moderate. Antineoplastic and immunomodulating agents showed a longer median TTO (14 days; moderate pattern).

In JADER, early-onset clustering was more pronounced. VARIOUS demonstrated an extreme acute pattern (median 0 days; 99.2% ≤ 7 days), while antiinfectives, dermatologicals, and respiratory system drugs also met strong criteria. In contrast, delayed patterns were observed for genito-urinary system drugs (median 28 days; weak) and anti-parkinson drugs (ATC Level 2; median 8.5 days; moderate).

At ATC Level 2, psycholeptics (N05) constituted the second-largest drug class in both databases (FAERS N = 2,924; JADER N = 339), with rapid onset in FAERS (median 1 day) but delayed onset in JADER (median 12 days). Antibacterials for systemic use (J01) exhibited strong acute signals in both databases (FAERS median 3 days, 74.0% ≤ 7 days; JADER median 2 days, 88.8% ≤ 7 days). Anesthetics (N01) also showed strong, concordant early-onset patterns. Analgesics (N02), although moderate in both databases, showed consistent median TTO of 2–5 days (Table 1).

**TABLE 1:** TTO, IQR, and signal strength of adverse events by drug class (L1 and L2) in FAERS and JADER.

Adverse effects	FAERS						JADER					
	N	IQR	Median (IQR)	≤7 days (%)	≤14 days (%)	Signal	N	IQR	Median (IQR)	≤7 days (%)	≤14 days (%)	Signal
ATC level 1												
Antineoplastic and immunomodulating agents	10,039	61.0	14.0 (2.0–63.0)	40.3%	50.7%	Moderate	605	40.0	12.0 (3.0–43.0)	43.6%	56.4%	Moderate
Nervous system	7,337	20.0	2.0 (0.0–20.0)	63.9%	71.5%	Moderate	874	38.0	10.0 (2.0–40.0)	46.7%	57.1%	Moderate
Antiinfectives for systemic use	3,007	10.0	3.0 (0.0–10.0)	69.1%	81.6%	Strong	375	9.0	3.0 (1.0–10.0)	72.3%	79.5%	Strong
Alimentary tract and metabolism	2,577	17.0	2.0 (0.0–17.0)	66.2%	73.7%	Moderate	361	38.0	12.0 (2.0–40.0)	43.2%	56.0%	Moderate
Cardiovascular system	1,584	50.0	8.0 (0.0–50.0)	49.5%	59.0%	Moderate	75	36.5	9.0 (3.0–39.5)	48.0%	53.3%	Moderate
Blood and blood forming organs	1,195	49.0	2.0 (0.0–49.0)	59.8%	64.4%	Moderate	37	24.0	10.0 (1.0–25.0)	48.6%	64.9%	Moderate
Dermatologicals	708	17.0	3.0 (0.0–17.0)	63.4%	72.5%	Moderate	165	7.0	3.0 (1.0–8.0)	73.3%	80.0%	Strong
Respiratory system	567	21.0	1.0 (0.0–21.0)	69.1%	73.7%	Moderate	38	5.8	2.0 (0.0–5.8)	78.9%	78.9%	Strong
Various	526	1.0	0.0 (0.0–1.0)	89.2%	90.9%	Strong	118	2.0	0.0 (0.0–2.0)	99.2%	100.0%	Strong
Musculo-skeletal system	498	15.0	2.0 (0.0–15.0)	67.1%	74.7%	Moderate	27	29.0	3.0 (1.0–30.0)	63.0%	63.0%	Moderate
Genito urinary system and sex hormones	346	15.0	2.0 (0.0–15.0)	65.6%	73.1%	Moderate	14	50.2	28.0 (17.2–67.5)	14.3%	21.4%	Weak
ATC level 2												
Antineoplastic agents	6,606	47.0	12.0 (2.0–49.0)	42.7%	54.5%	Moderate	495	40.0	9.0 (2.0–42.0)	46.9%	57.2%	Moderate
Psycholeptics	2,924	19.0	1.0 (0.0–19.0)	65.5%	73.0%	Moderate	339	56.0	12.0 (1.0–57.0)	44.2%	52.8%	Moderate
Immunosuppressants	2,692	101.0	22.0 (2.0–103.0)	35.6%	43.8%	Moderate	88	52.0	14.5 (7.8–59.8)	25.0%	50.0%	Moderate
Antibacterials for systemic use	1836	8.0	3.0 (0.0–8.0)	74.0%	87.4%	Strong	107	2.0	2.0 (1.0–3.0)	88.8%	90.7%	Strong
Analgesics	1,450	17.0	2.0 (0.0–17.0)	66.5%	73.4%	Moderate	141	15.0	5.0 (1.0–16.0)	61.0%	73.8%	Moderate
Psychoanaleptics	1,053	21.0	2.0 (0.0–21.0)	61.6%	69.6%	Moderate	100	51.8	16.5 (3.0–54.8)	41.0%	48.0%	Moderate
Antiepileptics	959	22.0	3.0 (0.0–22.0)	59.5%	68.4%	Moderate	157	48.0	16.0 (4.0–52.0)	35.7%	49.0%	Moderate
Antivirals for systemic use	803	28.0	6.0 (1.0–29.0)	52.9%	65.6%	Moderate	171	38.0	6.0 (2.0–40.0)	59.6%	66.1%	Moderate
Antithrombotic agents	651	112.0	16.0 (1.0–113.0)	43.2%	49.0%	Moderate	11	17.5	10.0 (1.5–19.0)	45.5%	72.7%	Moderate
Stomatological preparations	559	9.5	2.0 (0.0–9.5)	71.4%	82.8%	Strong	192	39.0	18.5 (6.0–45.0)	31.2%	45.8%	Moderate
Endocrine therapy	469	82.0	30.0 (6.0–88.0)	27.9%	34.8%	Weak	18	15.8	10.0 (6.2–22.0)	38.9%	61.1%	Moderate
Other dermatological preparations	398	39.0	5.0 (0.0–39.0)	52.8%	60.6%	Moderate	71	30.0	8.0 (3.0–33.0)	46.5%	56.3%	Moderate
Blood substitutes and perfusion solutions	390	0.0	0.0 (0.0–0.0)	95.6%	96.2%	Strong	16	17.8	5.0 (1.0–18.8)	68.8%	68.8%	Moderate
Other nervous system drugs	388	32.2	3.0 (0.0–32.2)	60.6%	64.7%	Moderate	39	34.5	12.0 (3.5–38.0)	48.7%	59.0%	Moderate
Anesthetics	372	11.5	1.0 (0.0–11.5)	69.9%	79.0%	Strong	68	13.0	5.0 (1.0–14.0)	61.8%	76.5%	Strong
Cardiac therapy	357	12.0	2.0 (0.0–12.0)	68.6%	79.0%	Strong	13	16.0	4.0 (1.0–17.0)	61.5%	69.2%	Moderate
Diuretics	346	53.8	10.5 (1.0–54.8)	42.2%	56.4%	Moderate	25	34.0	6.0 (1.0–35.0)	64.0%	68.0%	Moderate
Drugs for obstructive airway diseases	293	48.0	4.0 (0.0–48.0)	53.9%	56.7%	Moderate	32	10.2	3.0 (0.0–10.2)	75.0%	75.0%	Strong
Drugs for constipation	268	2.0	0.0 (0.0–2.0)	85.1%	85.8%	Strong	83	20.5	3.0 (0.0–20.5)	63.9%	73.5%	Moderate
Digestives, incl. enzymes	253	10.0	0.0 (0.0–10.0)	73.5%	77.9%	Strong	44	46.2	17.5 (6.2–52.5)	29.5%	47.7%	Moderate
Drugs for acid related disorders	225	16.0	3.0 (0.0–16.0)	66.2%	73.3%	Moderate	25	10.0	3.0 (2.0–12.0)	72.0%	76.0%	Strong
Anti-Parkinson drugs	191	50.5	7.0 (0.0–50.5)	50.8%	58.6%	Moderate	30	50.8	8.5 (2.8–53.5)	46.7%	53.3%	Moderate
Antimycotics for systemic use	187	3.0	2.0 (1.0–4.0)	83.4%	90.9%	Strong	84	2.2	2.0 (1.0–3.2)	85.7%	94.0%	Strong
Diagnostic agents	185	2.0	1.0 (0.0–2.0)	96.2%	98.4%	Strong	105	2.0	0.0 (0.0–2.0)	100.0%	100.0%	Strong
Antibiotics and chemotherapeutics for dermatological use	115	5.0	2.0 (1.0–6.0)	82.6%	93.0%	Strong	74	2.0	2.0 (1.0–3.0)	97.3%	100.0%	Strong
Antimycobacterials	58	32.8	12.0 (5.2–38.0)	37.9%	51.7%	Moderate	13	38.0	10.0 (10.0–48.0)	15.4%	69.2%	Moderate
Antiinflammatory and antirheumatic products	26	6.0	2.5 (0.0–6.0)	80.8%	80.8%	Strong	12	29.0	13.5 (1.0–30.0)	50.0%	50.0%	Moderate
Vasoprotectives	13	2.0	0.0 (0.0–2.0)	100.0%	100.0%	Strong	23	48.5	15.0 (6.0–54.5)	43.5%	43.5%	Moderate

**FIGURE 5:**
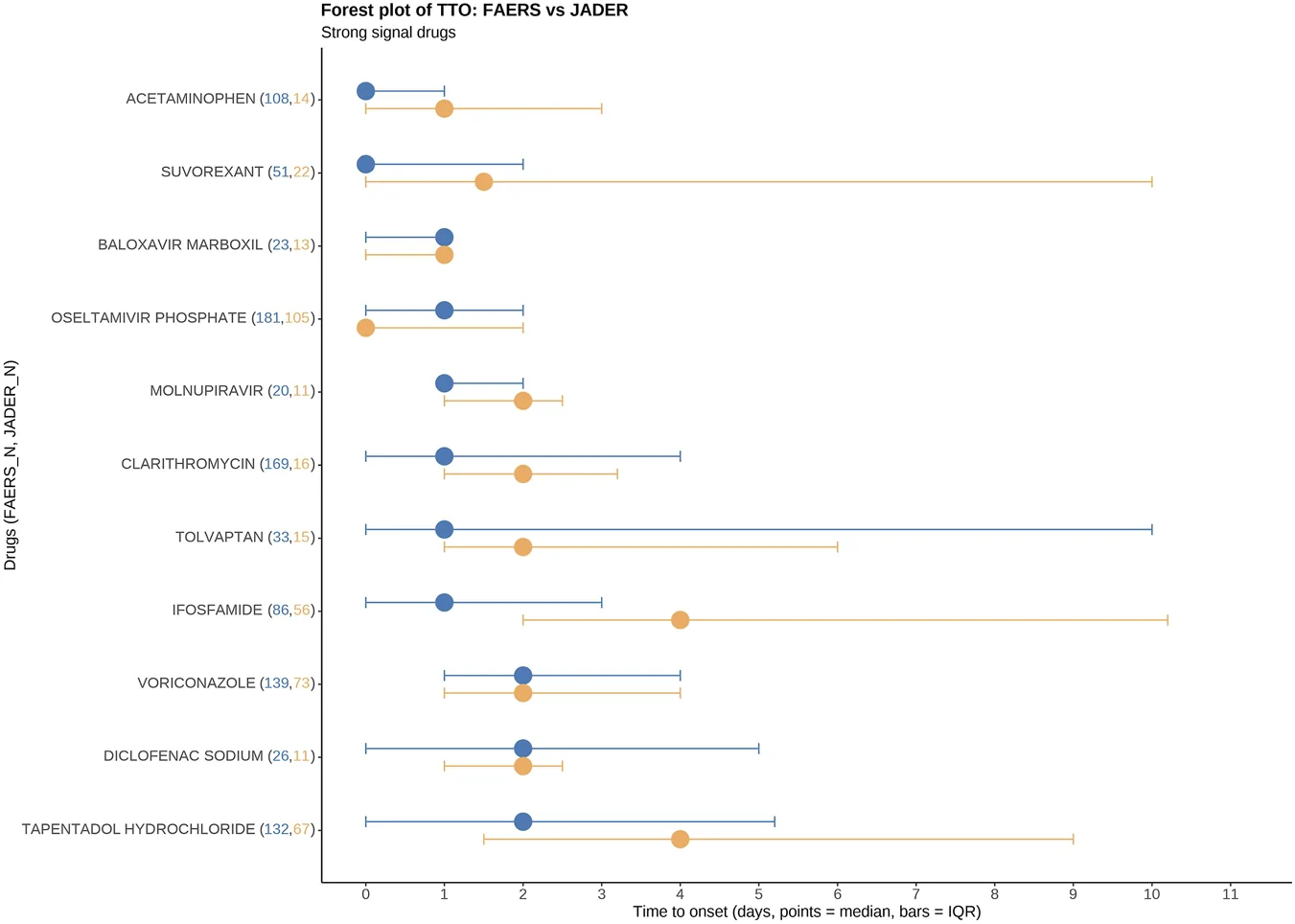
Forest plot of time-to-onset (TTO) patterns for selected drugs with Strong acute TTO patterns associated with delirium in FAERS and JADER. Points indicate median TTO; horizontal bars represent interquartile ranges (IQR).

Drug-level analysis (Figure 5; Supplementary Table S7) confirmed acute temporal clustering (Strong TTO pattern) in both databases for four priority agents, including oseltamivir phosphate (FAERS median 1 day, JADER median 0 day), clarithromycin (FAERS 1 day, JADER 2 days), voriconazole (both 2 days), and tapentadol hydrochloride (FAERS 2 days, JADER 4 days), with most cases occurring within 7 days of drug initiation.

## Discussion

This study presents a cross-national, comprehensive, multi-database pharmacovigilance analysis of drug-induced delirium, leveraging large-scale real-world data from FAERS, JADER, and CVAR databases. By integrating these three international databases, we were able to validate signals across regions, identify both shared and context-specific high-risk drugs, and provide a robust evidence framework for pharmacovigilance signal identification and regulatory decision-making. The establishment of structured post-marketing safety databases is critical for the timely identification and monitoring of drugs potentially associated with delirium, as emphasized in contemporary pharmacovigilance studies (Andronis et al., 2020; Chen et al., 2024).

Consistent with prior reports, our analyses confirmed that delirium disproportionately affects older adults, with sex-related differences varying across clinical settings and databases (Inouye et al., 2014; Ormseth et al., 2023). This sex difference may be partially explained by hormonal influences, genetic predispositions, or immune response variability, which have been reported in other neuropsychiatric ADRs. Age-related susceptibility is likely driven by polypharmacy, cumulative anticholinergic burden, reduced hepatic and renal clearance, neurodegenerative changes, and altered blood–brain barrier permeability, which collectively increase vulnerability to neurotoxicity (Moellm et al., 2025; Weidmann et al., 2025). Many high-risk drugs possess anticholinergic properties and are recognized as potentially inappropriate medications (PIMs) in older adults (By the American Geriatrics Society Beers Criteria Update Expert Panel, 2023; Salahudeen et al., 2015), highlighting the need for structured medication review and deprescribing strategies.

Centrally acting medications, opioids, systemic corticosteroids, immunomodulatory agents, and select anti-infectives showed variable signal patterns across databases. Psychotropic drugs, including benzodiazepines and antipsychotics, are well-recognized drugs associated with delirium (Bush et al., 2017). Mechanistically, these drugs may disrupt neurotransmitter balance through cholinergic deficiency, dopaminergic excess, and altered GABAergic signaling, all central to delirium pathophysiology. Benzodiazepines should be restricted mainly to withdrawal-related delirium management, as inappropriate use may constitute a drug-related problem of safety or necessity (Marcantonio, 2017). Opioids are associated with delirium reporting via central nervous system depression and modulation of pain-related neuroendocrine pathways; conversely, inadequate analgesia itself is a risk factor for delirium, emphasizing the complex balance between under- and overtreatment (Berger-Estilita and Bilotta, 2025; Leung et al., 2013).

Systemic corticosteroids are associated with delirium reporting through glucocorticoid-induced neurochemical alterations, including enhanced excitatory neurotransmission and HPA axis dysregulation. Anti-infectives, particularly those with central neurotoxicity or blood–brain barrier penetration, have been associated with delirium in prior reports (Alagiakrishnan and Wiens, 2004). Immunomodulatory agents show association with delirium indirectly via systemic inflammatory modulation, highlighting the interplay between peripheral immune activation and CNS dysfunction. Interestingly, drugs not previously suspected of being associated with delirium were also identified, reinforcing the need for vigilance across therapeutic categories (Andronis et al., 2020).

Additionally, our findings underscore the multifactorial etiology of delirium, driven by drug pharmacodynamics, host factors (age, sex, comorbidities), polypharmacy, and anticholinergic load. Higher cumulative anticholinergic burden is strongly associated with cognitive decline and increased morbidity (Taylor-Rowan et al., 2021; Gray et al., 2015). Structured pharmacotherapy review and deprescribing strategies are therefore critical, particularly in older adults and polypharmacy contexts.

Furthermore, TTO analyses revealed that delirium reporting is often highest within the first week after drug initiation, especially for psychotropic medications, reflecting an acute early-onset reporting profile. Drugs showing prolonged or delayed reporting patterns emphasize the need for ongoing vigilance throughout therapy. Early recognition within the first few days may allow timely intervention, potentially reducing complications such as falls, prolonged hospitalization, and functional decline (Szewczak et al., 2025). Proactive strategies should include careful medication review, dose adjustment, deprescribing, and avoidance of PIMs guided by AGS Beers Criteria and STOPP/START tools (By the American Geriatrics Society Beers Criteria Update Expert Panel, 2023; Lunghi et al., 2022).

The cross-database concordance of high-risk drug classes strengthens the robustness of observed signals and mitigates concerns regarding region-specific reporting bias. While passive surveillance is widely implemented and cost-effective, it is inherently limited by underreporting and variability in data quality (Zhang et al., 2023). Integrating active surveillance, electronic health records, and real-time signal detection can enhance both the sensitivity and specificity of pharmacovigilance monitoring (Nagar et al., 2025).

The database-specific signal profiles likely reflect differences in prescribing practices, regulatory environments, and reporting cultures across the three regions. For example, the predominance of anti-infective signals in JADER (e.g., valaciclovir, aciclovir) may reflect Japanese prescribing patterns for antiviral agents, whereas the immunomodulatory signals unique to CVAR (e.g., tocilizumab, abatacept) may relate to Canadian reporting patterns for biological therapies. Direct cross-database comparison of demographic distributions was deliberately avoided, as reporting volumes and patient characteristics reflect national spontaneous reporting frameworks rather than true epidemiological differences across regions.

However, several limitations still exist. First, spontaneous reporting systems are prone to underreporting, stimulated reporting, reporting bias, variable data quality and incomplete clinical data, precluding causal inference. Confounding factors including comorbidities, polypharmacy burden, anticholinergic burden, disease severity, and hospitalization context could not be fully adjusted. Second, heterogeneity in coding practices and reporting thresholds across databases may influence signal detection. Third, dose–response relationships, treatment duration, administration routes, and cumulative exposure could not be systematically analyzed. Fourth, delirium observed with immunomodulatory or CNS-active drugs may reflect underlying disease activity rather than direct drug effects. Fifth, while our 5-PT case definition captures the core clinical presentations of delirium and aligns with the predominant narrow-scope terms of the Noninfectious encephalopathy/delirium SMQ, it does not include all PTs within the SMQ; this focused approach was chosen to maximize specificity for drug-associated delirium. Sixth, 23.6% of FAERS TTO records (8,904/37,785) lacked ATC Level 1 classification mapping and were excluded from ATC-stratified TTO analyses; these records likely represent multi-ingredient products or drugs without established ATC codes.

Future research should prioritize prospective, longitudinal studies with standardized delirium assessment, systematic pharmacotherapy review, and integration of electronic health records. Individualized risk prediction using validated delirium prediction models (e.g., PRE-DELIRIC) (van den Boogaard et al., 2012) and anticholinergic load quantification (Carnahan et al., 2006), combined with mechanistic studies exploring neurotransmitter imbalance, neuroinflammation, and age-related pharmacokinetic/pharmacodynamic alterations, will enhance causal understanding and clinical management. The present findings should be interpreted as hypothesis-generating pharmacovigilance signals rather than confirmatory evidence of causal drug–delirium relationships.

## Conclusion

In conclusion, our multi-database pharmacovigilance study highlights the importance of early detection, structured pharmacotherapy review, and proactive monitoring of delirium in patients receiving high-risk medications. Older adults, particularly those exposed to polypharmacy and PIMs, remain at greatest risk. Institutional protocols integrating systematic adverse event reporting, medication review, deprescribing strategies, and risk-based monitoring are essential for mitigating drug-induced delirium and enhancing patient safety.

## Data Availability

The original contributions presented in the study are included in the article/Supplementary Material, further inquiries can be directed to the corresponding authors.
