# Supplementary material for: Cross national pharmacovigilance analysis of drug-induced delirium: a comparative study using the US FAERS, Japanese JADER, and canadian CVAR datasets
Source: Front Pharmacol. 2026 Sep 3;17:1878501. doi: 10.3389/fphar.2026.1878501 (PMC13582247; doi:10.3389/fphar.2026.1878501)
Supplement: Supplementary file 1 [file Supplementaryfile1.docx]

Supplementary Figure 1. Distribution of drug related delirium across FAERS, JADER, AND CVAR databases.

Supplementary Figure 2. Signal strength heatmap of drug-associated delirium across three pharmacovigilance databases (FAERS, JADER, Canada Vigilance).

****Color****: Signal strength based on the lower bound of the 95% confidence interval，**Star**: Formal positive signal

Supplementary Table 1. Disproportionality Analysis Positive Signals from FAERS, JADER, and CVAR Databases

| **Category** | **Name** | **FAERS** | **JADER** | **CVAR** |
| --- | --- | --- | --- | --- |
| **Drugs** | PREGABALIN | ✓ | ✓ | ✓ |
|  | DULOXETINE HYDROCHLORIDE | ✓ | ✓ |  |
|  | LAMOTRIGINE | ✓ | ✓ |  |
|  | QUETIAPINE | ✓ |  | ✓ |
|  | QUETIAPINE FUMARATE | ✓ | ✓ |  |
|  | ZOLPIDEM TARTRATE | ✓ | ✓ |  |
|  | ABATACEPT |  |  | ✓ |
|  | ACETAMINOPHEN |  |  | ✓ |
|  | ACICLOVIR |  | ✓ |  |
|  | ALENDRONATE |  |  | ✓ |
|  | ALPRAZOLAM | ✓ |  |  |
|  | AMANTADINE HYDROCHLORIDE |  | ✓ |  |
|  | AMLODIPINE BESYLATE | ✓ |  |  |
|  | APREMILAST |  |  | ✓ |
|  | ARIPIPRAZOLE | ✓ |  |  |
|  | ASCORBIC ACID |  |  | ✓ |
|  | ASPIRIN |  |  | ✓ |
|  | BACLOFEN | ✓ |  |  |
|  | BROTIZOLAM |  | ✓ |  |
|  | CALCIUM CARBONATE |  |  | ✓ |
|  | CANDESARTAN |  |  | ✓ |
|  | CARBIDOPA\LEVODOPA | ✓ |  |  |
|  | CEFEPIME DIHYDROCHLORIDE HYDRATE |  | ✓ |  |
|  | CELECOXIB |  |  | ✓ |
|  | Certolizumab Pegol |  |  | ✓ |
|  | Cetirizine |  |  | ✓ |
|  | Chlorhexidine |  |  | ✓ |
|  | CHOLECALCIFEROL |  |  | ✓ |
|  | CIPROFLOXACIN | ✓ |  |  |
|  | CORTISONE |  |  | ✓ |
|  | DESOXIMETASONE |  |  | ✓ |
|  | Diclofenac |  |  | ✓ |
|  | DONEPEZIL HYDROCHLORIDE |  | ✓ |  |
|  | ETIZOLAM |  | ✓ |  |
|  | FAMOTIDINE |  | ✓ |  |
|  | FENTANYL |  | ✓ |  |
|  | FENTANYL CITRATE |  | ✓ |  |
|  | FLUMETHASONE |  |  | ✓ |
|  | FLUNITRAZEPAM |  | ✓ |  |
|  | FOLIC ACID |  |  | ✓ |
|  | FUROSEMIDE | ✓ |  |  |
|  | GABAPENTIN | ✓ |  |  |
|  | GOLIMUMAB |  |  | ✓ |
|  | Hydrocortisone |  |  | ✓ |
|  | HYDROMORPHONE |  |  | ✓ |
|  | Hydroxychloroquine |  |  | ✓ |
|  | IFOSFAMIDE |  | ✓ |  |
|  | LEFLUNOMIDE |  |  | ✓ |
|  | LEVETIRACETAM | ✓ |  |  |
|  | LEVOFLOXACIN | ✓ |  |  |
|  | LORAZEPAM | ✓ |  |  |
|  | Metformin |  |  | ✓ |
|  | METFORMIN HYDROCHLORIDE | ✓ |  |  |
|  | METHYLPREDNISOLONE |  |  | ✓ |
|  | METRONIDAZOLE |  | ✓ |  |
|  | MIDAZOLAM |  | ✓ |  |
|  | MIRTAZAPINE |  | ✓ |  |
|  | Morphine |  |  | ✓ |
|  | MORPHINE HYDROCHLORIDE HYDRATE |  | ✓ |  |
|  | NAPROXEN |  |  | ✓ |
|  | OCTREOTIDE |  |  | ✓ |
|  | OLANZAPINE | ✓ |  |  |
|  | OSELTAMIVIR PHOSPHATE |  | ✓ |  |
|  | Oxycodone |  |  | ✓ |
|  | OXYCODONE HYDROCHLORIDE HYDRATE |  | ✓ |  |
|  | PANTOPRAZOLE |  |  | ✓ |
|  | PAROXETINE HYDROCHLORIDE HYDRATE |  | ✓ |  |
|  | PHTHALYLSULFATHIAZOLE |  |  | ✓ |
|  | PIMAVANSERIN TARTRATE | ✓ |  |  |
|  | PRAMIPEXOLE HYDROCHLORIDE HYDRATE |  | ✓ |  |
|  | Prednisone |  |  | ✓ |
|  | Pseudoephedrine |  |  | ✓ |
|  | RAMIPRIL |  |  | ✓ |
|  | RECOMBINANT ADSORBED BIVALENT HUMAN PAPILLOMAVIRUS VLP VACCINE (INSECT CELL) |  | ✓ |  |
|  | RIVASTIGMINE |  | ✓ |  |
|  | SARILUMAB |  |  | ✓ |
|  | SERTRALINE HYDROCHLORIDE | ✓ |  |  |
|  | SULFAMETHOXAZOLE |  |  | ✓ |
|  | SULFASALAZINE |  |  | ✓ |
|  | SUVOREXANT |  | ✓ |  |
|  | TAPENTADOL HYDROCHLORIDE |  | ✓ |  |
|  | THEOPHYLLINE |  | ✓ |  |
|  | TOCILIZUMAB |  |  | ✓ |
|  | Tofacitinib |  |  | ✓ |
|  | TRAMADOL | ✓ |  |  |
|  | TRAMADOL HYDROCHLORIDE/ACETAMINOPHEN COMBINATION |  | ✓ |  |
|  | USTEKINUMAB |  |  | ✓ |
|  | VALACICLOVIR HYDROCHLORIDE |  | ✓ |  |
|  | VENLAFAXINE HYDROCHLORIDE | ✓ |  |  |
|  | VORICONAZOLE |  | ✓ |  |
|  | ZANAMIVIR HYDRATE |  | ✓ |  |
| **ATC Level 1** | N NERVOUS SYSTEM | ✓ | ✓ | ✓ |
|  | B BLOOD AND BLOOD FORMING ORGANS |  |  | ✓ |
|  | D DERMATOLOGICALS |  |  | ✓ |
|  | H SYSTEMIC HORMONAL PREPARATIONS, EXCL. SEX HORMONES AND INSULINS |  |  | ✓ |
|  | J ANTIINFECTIVES FOR SYSTEMIC USE |  |  | ✓ |
|  | R RESPIRATORY SYSTEM |  | ✓ |  |
| **ATC Level 2** | N02 ANALGESICS | ✓ | ✓ | ✓ |
|  | N05 PSYCHOLEPTICS | ✓ | ✓ | ✓ |
|  | N06 PSYCHOANALEPTICS | ✓ | ✓ | ✓ |
|  | J01 ANTIBACTERIALS FOR SYSTEMIC USE | ✓ |  | ✓ |
|  | A01 STOMATOLOGICAL PREPARATIONS |  |  | ✓ |
|  | A06 DRUGS FOR CONSTIPATION |  | ✓ |  |
|  | D11 OTHER DERMATOLOGICAL PREPARATIONS |  |  | ✓ |
|  | N03 ANTIEPILEPTICS | ✓ |  |  |

Supplementary Table 2. Disproportionality signals for drug-associated delirium in FAERS: Top 50 drugs and top 10 ATC classifications at Level 1 (L1) and Level 2 (L2)

| **drugs** | **cases** | **ROR** | **ROR_lower** | **ROR_upper** | **PRR** | **PRR_lower** | **PRR_upper** | **IC** | **IC_lower** | **EBGM** | **EBGM_lower** | **ROR_positive** | **PRR_positive** | **IC_positive** | **EBGM_positive** | **overall_positive** |
| --- | --- | --- | --- | --- | --- | --- | --- | --- | --- | --- | --- | --- | --- | --- | --- | --- |
| BACLOFEN | 2149 | 12 | 11.47 | 12.55 | 10.7 | 10.28 | 11.14 | 3.4 | 3.35 | 10.57 | 10.2 | Yes | Yes | Yes | Yes | Positive |
| DULOXETINE HYDROCHLORIDE | 2753 | 9.57 | 9.2 | 9.96 | 8.75 | 8.44 | 9.07 | 3.11 | 3.06 | 8.62 | 8.35 | Yes | Yes | Yes | Yes | Positive |
| PIMAVANSERIN TARTRATE | 4252 | 9.33 | 9.04 | 9.63 | 8.55 | 8.31 | 8.81 | 3.06 | 3.02 | 8.36 | 8.15 | Yes | Yes | Yes | Yes | Positive |
| LORAZEPAM | 779 | 6.41 | 5.96 | 6.89 | 6.04 | 5.65 | 6.47 | 2.59 | 2.49 | 6.01 | 5.66 | Yes | Yes | Yes | Yes | Positive |
| TRAMADOL | 746 | 5.79 | 5.37 | 6.23 | 5.5 | 5.13 | 5.89 | 2.45 | 2.35 | 5.47 | 5.15 | Yes | Yes | Yes | Yes | Positive |
| ZOLPIDEM TARTRATE | 678 | 5.5 | 5.09 | 5.95 | 5.24 | 4.87 | 5.64 | 2.39 | 2.28 | 5.22 | 4.9 | Yes | Yes | Yes | Yes | Positive |
| QUETIAPINE | 1237 | 5.33 | 5.03 | 5.64 | 5.08 | 4.81 | 5.37 | 2.34 | 2.26 | 5.05 | 4.82 | Yes | Yes | Yes | Yes | Positive |
| OLANZAPINE | 1440 | 4.72 | 4.48 | 4.98 | 4.53 | 4.31 | 4.77 | 2.17 | 2.1 | 4.5 | 4.31 | Yes | Yes | Yes | Yes | Positive |
| QUETIAPINE FUMARATE | 737 | 4.7 | 4.37 | 5.07 | 4.52 | 4.21 | 4.85 | 2.17 | 2.07 | 4.49 | 4.22 | Yes | Yes | Yes | Yes | Positive |
| CARBIDOPA\LEVODOPA | 2377 | 4.1 | 3.94 | 4.28 | 3.97 | 3.81 | 4.13 | 1.97 | 1.92 | 3.92 | 3.79 | Yes | Yes | Yes | Yes | Positive |
| GABAPENTIN | 2070 | 4.04 | 3.87 | 4.22 | 3.91 | 3.75 | 4.08 | 1.95 | 1.89 | 3.87 | 3.73 | Yes | Yes | Yes | Yes | Positive |
| ALPRAZOLAM | 889 | 3.79 | 3.54 | 4.05 | 3.67 | 3.45 | 3.92 | 1.87 | 1.78 | 3.65 | 3.45 | Yes | Yes | Yes | Yes | Positive |
| FUROSEMIDE | 720 | 3.56 | 3.3 | 3.83 | 3.46 | 3.22 | 3.71 | 1.79 | 1.68 | 3.43 | 3.23 | Yes | Yes | Yes | Yes | Positive |
| VENLAFAXINE HYDROCHLORIDE | 986 | 3.47 | 3.25 | 3.7 | 3.38 | 3.17 | 3.59 | 1.75 | 1.66 | 3.35 | 3.18 | Yes | Yes | Yes | Yes | Positive |
| SERTRALINE HYDROCHLORIDE | 1287 | 3.43 | 3.24 | 3.63 | 3.34 | 3.16 | 3.52 | 1.73 | 1.66 | 3.31 | 3.17 | Yes | Yes | Yes | Yes | Positive |
| LAMOTRIGINE | 994 | 3.25 | 3.05 | 3.46 | 3.17 | 2.98 | 3.37 | 1.66 | 1.57 | 3.15 | 2.99 | Yes | Yes | Yes | Yes | Positive |
| CIPROFLOXACIN | 721 | 3.21 | 2.98 | 3.46 | 3.14 | 2.92 | 3.37 | 1.65 | 1.54 | 3.11 | 2.93 | Yes | Yes | Yes | Yes | Positive |
| LEVOFLOXACIN | 790 | 2.94 | 2.74 | 3.16 | 2.88 | 2.69 | 3.08 | 1.52 | 1.42 | 2.86 | 2.7 | Yes | Yes | Yes | Yes | Positive |
| LEVETIRACETAM | 1400 | 2.87 | 2.72 | 3.03 | 2.81 | 2.67 | 2.96 | 1.49 | 1.41 | 2.79 | 2.67 | Yes | Yes | Yes | Yes | Positive |
| PREGABALIN | 2110 | 2.66 | 2.55 | 2.78 | 2.61 | 2.5 | 2.72 | 1.37 | 1.31 | 2.59 | 2.49 | Yes | Yes | Yes | Yes | Positive |
| METFORMIN HYDROCHLORIDE | 1524 | 2.54 | 2.41 | 2.67 | 2.49 | 2.37 | 2.62 | 1.31 | 1.24 | 2.47 | 2.37 | Yes | Yes | Yes | Yes | Positive |
| AMLODIPINE BESYLATE | 863 | 2.16 | 2.02 | 2.32 | 2.14 | 2 | 2.28 | 1.09 | 1 | 2.13 | 2.01 | Yes | Yes | Yes | Yes | Positive |
| ARIPIPRAZOLE | 1088 | 2.04 | 1.92 | 2.17 | 2.02 | 1.91 | 2.14 | 1.01 | 0.93 | 2.01 | 1.91 | Yes | Yes | Yes | Yes | Positive |
| NATALIZUMAB | 1299 | 1.96 | 1.85 | 2.07 | 1.94 | 1.84 | 2.05 | 0.95 | 0.87 | 1.93 | 1.84 | No | No | Yes | No | Negative |
| SODIUM OXYBATE | 1283 | 1.95 | 1.85 | 2.06 | 1.93 | 1.83 | 2.04 | 0.94 | 0.87 | 1.92 | 1.84 | No | No | Yes | No | Negative |
| ENZALUTAMIDE | 921 | 1.95 | 1.82 | 2.08 | 1.93 | 1.81 | 2.05 | 0.94 | 0.85 | 1.92 | 1.81 | No | No | Yes | No | Negative |
| FENTANYL | 721 | 1.93 | 1.79 | 2.08 | 1.91 | 1.78 | 2.05 | 0.93 | 0.83 | 1.9 | 1.79 | No | No | Yes | No | Negative |
| DEXAMETHASONE | 695 | 1.68 | 1.56 | 1.81 | 1.67 | 1.55 | 1.79 | 0.74 | 0.63 | 1.66 | 1.56 | No | No | Yes | No | Negative |
| CLOZAPINE | 1447 | 1.62 | 1.54 | 1.71 | 1.61 | 1.53 | 1.7 | 0.69 | 0.61 | 1.61 | 1.54 | No | No | Yes | No | Negative |
| INTERFERON BETA-1A | 1092 | 1.58 | 1.49 | 1.68 | 1.57 | 1.48 | 1.67 | 0.65 | 0.56 | 1.57 | 1.49 | No | No | Yes | No | Negative |
| TACROLIMUS | 759 | 1.54 | 1.43 | 1.65 | 1.53 | 1.42 | 1.64 | 0.61 | 0.51 | 1.52 | 1.44 | No | No | Yes | No | Negative |
| DIMETHYL FUMARATE | 1385 | 1.48 | 1.4 | 1.56 | 1.47 | 1.4 | 1.55 | 0.55 | 0.48 | 1.47 | 1.4 | No | No | Yes | No | Negative |
| RISPERIDONE | 940 | 1.44 | 1.35 | 1.54 | 1.43 | 1.34 | 1.53 | 0.52 | 0.43 | 1.43 | 1.35 | No | No | Yes | No | Negative |
| ACETAMINOPHEN | 1195 | 1.43 | 1.35 | 1.51 | 1.42 | 1.34 | 1.5 | 0.5 | 0.42 | 1.42 | 1.35 | No | No | Yes | No | Negative |
| METHOTREXATE | 1093 | 1.35 | 1.27 | 1.43 | 1.34 | 1.27 | 1.43 | 0.43 | 0.34 | 1.34 | 1.28 | No | No | Yes | No | Negative |
| FINGOLIMOD HYDROCHLORIDE | 957 | 1.33 | 1.25 | 1.42 | 1.32 | 1.24 | 1.41 | 0.4 | 0.31 | 1.32 | 1.25 | No | No | Yes | No | Negative |
| NIVOLUMAB | 901 | 1.23 | 1.15 | 1.31 | 1.22 | 1.15 | 1.31 | 0.29 | 0.2 | 1.22 | 1.16 | No | No | Yes | No | Negative |
| OXYCODONE HYDROCHLORIDE | 1479 | 1.09 | 1.04 | 1.15 | 1.09 | 1.04 | 1.15 | 0.12 | 0.05 | 1.09 | 1.04 | No | No | Yes | No | Negative |
| TREPROSTINIL | 947 | 0.93 | 0.87 | 0.99 | 0.93 | 0.87 | 0.99 | -0.1 | -0.2 | 0.93 | 0.88 | No | No | No | No | Negative |
| RITUXIMAB | 838 | 0.89 | 0.84 | 0.96 | 0.9 | 0.84 | 0.96 | -0.16 | -0.25 | 0.9 | 0.85 | No | No | No | No | Negative |
| SACUBITRIL\VALSARTAN | 978 | 0.84 | 0.78 | 0.89 | 0.84 | 0.79 | 0.89 | -0.25 | -0.34 | 0.84 | 0.79 | No | No | No | No | Negative |
| POMALIDOMIDE | 739 | 0.81 | 0.76 | 0.87 | 0.81 | 0.76 | 0.88 | -0.29 | -0.4 | 0.82 | 0.76 | No | No | No | No | Negative |
| PALBOCICLIB | 707 | 0.8 | 0.74 | 0.86 | 0.8 | 0.74 | 0.86 | -0.32 | -0.42 | 0.8 | 0.75 | No | No | No | No | Negative |
| LENALIDOMIDE | 2109 | 0.6 | 0.57 | 0.63 | 0.6 | 0.58 | 0.63 | -0.72 | -0.78 | 0.61 | 0.59 | No | No | No | No | Negative |
| TOFACITINIB CITRATE | 799 | 0.55 | 0.52 | 0.59 | 0.56 | 0.52 | 0.6 | -0.84 | -0.94 | 0.56 | 0.53 | No | No | No | No | Negative |
| APIXABAN | 769 | 0.49 | 0.45 | 0.52 | 0.49 | 0.46 | 0.53 | -1.02 | -1.12 | 0.49 | 0.47 | No | No | No | No | Negative |
| SECUKINUMAB | 711 | 0.48 | 0.44 | 0.51 | 0.48 | 0.44 | 0.51 | -1.06 | -1.16 | 0.48 | 0.45 | No | No | No | No | Negative |
| ADALIMUMAB | 1890 | 0.38 | 0.37 | 0.4 | 0.39 | 0.37 | 0.4 | -1.35 | -1.41 | 0.39 | 0.38 | No | No | No | No | Negative |
| ETANERCEPT | 735 | 0.22 | 0.2 | 0.23 | 0.22 | 0.2 | 0.24 | -2.16 | -2.26 | 0.22 | 0.21 | No | No | No | No | Negative |
| DUPILUMAB | 697 | 0.16 | 0.15 | 0.18 | 0.17 | 0.15 | 0.18 | -2.56 | -2.67 | 0.17 | 0.16 | No | No | No | No | Negative |
| **ATC L1** |  |  |  |  |  |  |  |  |  |  |  |  |  |  |  |  |
| ALIMENTARY TRACT AND METABOLISM | 12709 | 0.97 | 0.95 | 0.99 | 0.97 | 0.95 | 0.99 | -0.04 | -0.06 | 0.97 | 0.96 | No | No | No | No | Negative |
| BLOOD AND BLOOD FORMING ORGANS | 4883 | 0.68 | 0.66 | 0.7 | 0.68 | 0.66 | 0.7 | -0.54 | -0.58 | 0.69 | 0.67 | No | No | No | No | Negative |
| CARDIOVASCULAR SYSTEM | 8853 | 1.01 | 0.98 | 1.03 | 1.01 | 0.98 | 1.03 | 0.01 | -0.02 | 1 | 0.99 | No | No | No | No | Negative |
| DERMATOLOGICALS | 4226 | 0.41 | 0.39 | 0.42 | 0.41 | 0.4 | 0.42 | -1.24 | -1.28 | 0.42 | 0.41 | No | No | No | No | Negative |
| SYSTEMIC HORMONAL PREPARATIONS, EXCL. SEX HORMONES AND INSULINS | 2221 | 0.72 | 0.69 | 0.75 | 0.72 | 0.69 | 0.75 | -0.47 | -0.53 | 0.72 | 0.7 | No | No | No | No | Negative |
| ANTIINFECTIVES FOR SYSTEMIC USE | 6706 | 1.4 | 1.37 | 1.44 | 1.4 | 1.36 | 1.43 | 0.47 | 0.43 | 1.38 | 1.35 | No | No | Yes | No | Negative |
| ANTINEOPLASTIC AND IMMUNOMODULATING AGENTS | 40573 | 0.65 | 0.64 | 0.65 | 0.65 | 0.64 | 0.66 | -0.44 | -0.46 | 0.73 | 0.73 | No | No | No | No | Negative |
| MUSCULO-SKELETAL SYSTEM | 3558 | 0.93 | 0.9 | 0.97 | 0.93 | 0.9 | 0.97 | -0.1 | -0.14 | 0.94 | 0.91 | No | No | No | No | Negative |
| NERVOUS SYSTEM | 37408 | 2.99 | 2.96 | 3.03 | 2.94 | 2.9 | 2.97 | 1.32 | 1.31 | 2.5 | 2.48 | Yes | Yes | Yes | Yes | Positive |
| RESPIRATORY SYSTEM | 1765 | 0.58 | 0.55 | 0.61 | 0.58 | 0.55 | 0.61 | -0.77 | -0.84 | 0.59 | 0.56 | No | No | No | No | Negative |
| **ATC L2** |  |  |  |  |  |  |  |  |  |  |  |  |  |  |  |  |
| DRUGS USED IN DIABETES | 3566 | 0.49 | 0.48 | 0.51 | 0.49 | 0.48 | 0.51 | -0.99 | -1.03 | 0.5 | 0.49 | No | No | No | No | Negative |
| ANTITHROMBOTIC AGENTS | 3580 | 0.68 | 0.66 | 0.7 | 0.68 | 0.66 | 0.7 | -0.54 | -0.59 | 0.69 | 0.67 | No | No | No | No | Negative |
| OTHER DERMATOLOGICAL PREPARATIONS | 2784 | 0.42 | 0.41 | 0.44 | 0.42 | 0.41 | 0.44 | -1.21 | -1.26 | 0.43 | 0.42 | No | No | No | No | Negative |
| ANTIBACTERIALS FOR SYSTEMIC USE | 3746 | 2.09 | 2.02 | 2.16 | 2.07 | 2 | 2.13 | 1.03 | 0.99 | 2.04 | 1.99 | Yes | Yes | Yes | Yes | Positive |
| ANTINEOPLASTIC AGENTS | 18912 | 1.02 | 1 | 1.03 | 1.02 | 1 | 1.03 | 0.02 | 0 | 1.01 | 1 | No | No | Yes | No | Negative |
| IMMUNOSUPPRESSANTS | 17622 | 0.5 | 0.49 | 0.5 | 0.5 | 0.49 | 0.51 | -0.86 | -0.88 | 0.55 | 0.54 | No | No | No | No | Negative |
| ANALGESICS | 6971 | 2.18 | 2.12 | 2.23 | 2.15 | 2.1 | 2.2 | 1.07 | 1.04 | 2.1 | 2.06 | Yes | Yes | Yes | Yes | Positive |
| ANTIEPILEPTICS | 5863 | 2.8 | 2.72 | 2.87 | 2.74 | 2.67 | 2.82 | 1.42 | 1.39 | 2.68 | 2.62 | Yes | Yes | Yes | Yes | Positive |
| PSYCHOLEPTICS | 14993 | 3.64 | 3.58 | 3.7 | 3.54 | 3.48 | 3.6 | 1.73 | 1.71 | 3.31 | 3.27 | Yes | Yes | Yes | Yes | Positive |
| PSYCHOANALEPTICS | 4525 | 3.58 | 3.47 | 3.69 | 3.48 | 3.38 | 3.58 | 1.77 | 1.73 | 3.41 | 3.33 | Yes | Yes | Yes | Yes | Positive |

Supplementary Table 3. Disproportionality signals for drug-associated delirium in JADER: Top 50 drugs and top 10 ATC classifications at Level 1 (L1) and Level 2 (L2)

| **durgs** | **cases** | **ROR** | **ROR_lower** | **ROR_upper** | **PRR** | **PRR_lower** | **PRR_upper** | **IC** | **IC_lower** | **EBGM** | **EBGM_lower** | **ROR_positive** | **PRR_positive** | **IC_positive** | **EBGM_positive** | **overall_positive** |
| --- | --- | --- | --- | --- | --- | --- | --- | --- | --- | --- | --- | --- | --- | --- | --- | --- |
| Aciclovir | 199 | 14.87 | 12.84 | 17.23 | 13.53 | 11.85 | 15.46 | 3.74 | 3.55 | 13.25 | 11.77 | Yes | Yes | Yes | Yes | Positive |
| Amantadine Hydrochloride | 109 | 12.01 | 9.87 | 14.62 | 11.13 | 9.29 | 13.34 | 3.47 | 3.21 | 10.91 | 9.3 | Yes | Yes | Yes | Yes | Positive |
| Aripiprazole | 65 | 1.26 | 0.98 | 1.61 | 1.26 | 0.98 | 1.6 | 0.34 | -0.01 | 1.24 | 1 | No | No | No | No | Negative |
| Ifosfamide | 77 | 5.99 | 4.77 | 7.54 | 5.79 | 4.65 | 7.2 | 2.54 | 2.22 | 5.67 | 4.66 | Yes | Yes | Yes | Yes | Positive |
| Etizolam | 107 | 5.61 | 4.62 | 6.81 | 5.43 | 4.51 | 6.55 | 2.44 | 2.17 | 5.33 | 4.52 | Yes | Yes | Yes | Yes | Positive |
| Oxycodone Hydrochloride Hydrate | 419 | 33.73 | 30.28 | 37.57 | 27.41 | 25.11 | 29.92 | 4.74 | 4.62 | 26.46 | 24.4 | Yes | Yes | Yes | Yes | Positive |
| Oseltamivir Phosphate | 136 | 6.39 | 5.38 | 7.59 | 6.15 | 5.21 | 7.26 | 2.61 | 2.38 | 6.05 | 5.24 | Yes | Yes | Yes | Yes | Positive |
| Quetiapine Fumarate | 105 | 2.92 | 2.4 | 3.54 | 2.88 | 2.38 | 3.48 | 1.52 | 1.25 | 2.78 | 2.36 | Yes | Yes | Yes | Yes | Positive |
| Clozapine | 166 | 1.28 | 1.1 | 1.5 | 1.28 | 1.1 | 1.49 | 0.36 | 0.14 | 1.27 | 1.12 | No | No | Yes | No | Negative |
| SARS-CoV-2 RNA Vaccine | 194 | 1.01 | 0.88 | 1.17 | 1.01 | 0.88 | 1.16 | 0.02 | -0.18 | 1.01 | 0.9 | No | No | No | No | Negative |
| Zanamivir Hydrate | 91 | 14.69 | 11.83 | 18.24 | 13.37 | 10.99 | 16.26 | 3.74 | 3.46 | 13.06 | 10.95 | Yes | Yes | Yes | Yes | Positive |
| Ciclosporin | 109 | 1.09 | 0.9 | 1.31 | 1.09 | 0.9 | 1.31 | 0.13 | -0.14 | 1.08 | 0.92 | No | No | No | No | Negative |
| Suvorexant | 75 | 12.99 | 10.25 | 16.47 | 11.96 | 9.62 | 14.85 | 3.58 | 3.27 | 11.67 | 9.61 | Yes | Yes | Yes | Yes | Positive |
| Cefepime Dihydrochloride Hydrate | 84 | 9.32 | 7.46 | 11.63 | 8.79 | 7.14 | 10.82 | 3.14 | 2.84 | 8.62 | 7.18 | Yes | Yes | Yes | Yes | Positive |
| Zolpidem Tartrate | 261 | 12.04 | 10.6 | 13.67 | 11.16 | 9.92 | 12.55 | 3.46 | 3.29 | 10.92 | 9.85 | Yes | Yes | Yes | Yes | Positive |
| Tacrolimus Hydrate | 195 | 1.07 | 0.92 | 1.23 | 1.07 | 0.93 | 1.23 | 0.09 | -0.11 | 1.06 | 0.94 | No | No | No | No | Negative |
| Tapentadol Hydrochloride | 114 | 41.76 | 33.84 | 51.52 | 32.29 | 27.47 | 37.96 | 5.01 | 4.78 | 31.09 | 26.58 | Yes | Yes | Yes | Yes | Positive |
| Theophylline | 122 | 15.5 | 12.85 | 18.7 | 14.04 | 11.85 | 16.62 | 3.81 | 3.56 | 13.74 | 11.81 | Yes | Yes | Yes | Yes | Positive |
| Dexamethasone | 241 | 1.79 | 1.58 | 2.04 | 1.78 | 1.57 | 2.02 | 0.83 | 0.65 | 1.76 | 1.58 | No | No | Yes | No | Negative |
| Duloxetine Hydrochloride | 119 | 5.77 | 4.8 | 6.94 | 5.58 | 4.68 | 6.66 | 2.48 | 2.22 | 5.48 | 4.69 | Yes | Yes | Yes | Yes | Positive |
| Tramadol Hydrochloride/Acetaminophen Combination | 126 | 6.89 | 5.76 | 8.25 | 6.61 | 5.57 | 7.85 | 2.72 | 2.48 | 6.51 | 5.6 | Yes | Yes | Yes | Yes | Positive |
| Donepezil Hydrochloride | 110 | 4.55 | 3.76 | 5.51 | 4.44 | 3.69 | 5.34 | 2.15 | 1.89 | 4.33 | 3.68 | Yes | Yes | Yes | Yes | Positive |
| Nivolumab (Genetical Recombination) | 112 | 0.5 | 0.41 | 0.6 | 0.5 | 0.41 | 0.6 | -0.98 | -1.25 | 0.51 | 0.43 | No | No | No | No | Negative |
| Valaciclovir Hydrochloride | 1275 | 47.11 | 44.14 | 50.29 | 36.08 | 34.3 | 37.96 | 5.05 | 4.98 | 32.93 | 31.44 | Yes | Yes | Yes | Yes | Positive |
| Sodium Valproate | 80 | 1.95 | 1.57 | 2.44 | 1.94 | 1.56 | 2.41 | 0.96 | 0.65 | 1.89 | 1.56 | No | No | Yes | No | Negative |
| Paroxetine Hydrochloride Hydrate | 173 | 4.53 | 3.89 | 5.27 | 4.41 | 3.81 | 5.12 | 2.13 | 1.92 | 4.32 | 3.8 | Yes | Yes | Yes | Yes | Positive |
| Famotidine | 125 | 4.1 | 3.43 | 4.9 | 4.01 | 3.37 | 4.76 | 2 | 1.75 | 3.9 | 3.35 | Yes | Yes | Yes | Yes | Positive |
| Fentanyl | 163 | 14.67 | 12.47 | 17.25 | 13.36 | 11.53 | 15.47 | 3.73 | 3.52 | 13.09 | 11.49 | Yes | Yes | Yes | Yes | Positive |
| Fentanyl Citrate | 115 | 5.8 | 4.81 | 6.99 | 5.61 | 4.68 | 6.71 | 2.48 | 2.23 | 5.51 | 4.7 | Yes | Yes | Yes | Yes | Positive |
| Fluorouracil | 122 | 0.44 | 0.37 | 0.53 | 0.45 | 0.37 | 0.53 | -1.14 | -1.4 | 0.45 | 0.39 | No | No | No | No | Negative |
| Flunitrazepam | 69 | 4.06 | 3.19 | 5.16 | 3.97 | 3.14 | 5.01 | 1.99 | 1.66 | 3.81 | 3.09 | Yes | Yes | Yes | Yes | Positive |
| Brotizolam | 105 | 8.01 | 6.57 | 9.76 | 7.62 | 6.33 | 9.19 | 2.93 | 2.66 | 7.5 | 6.37 | Yes | Yes | Yes | Yes | Positive |
| Pramipexole Hydrochloride Hydrate | 111 | 11.34 | 9.34 | 13.77 | 10.55 | 8.82 | 12.63 | 3.4 | 3.14 | 10.35 | 8.83 | Yes | Yes | Yes | Yes | Positive |
| Pregabalin | 325 | 5.12 | 4.58 | 5.73 | 4.98 | 4.47 | 5.54 | 2.29 | 2.14 | 4.86 | 4.43 | Yes | Yes | Yes | Yes | Positive |
| Prednisolone | 204 | 0.64 | 0.55 | 0.73 | 0.64 | 0.56 | 0.73 | -0.63 | -0.83 | 0.64 | 0.57 | No | No | No | No | Negative |
| Peginterferon Alfa-2b (Genetical Recombination) | 77 | 0.86 | 0.69 | 1.08 | 0.86 | 0.69 | 1.08 | -0.21 | -0.53 | 0.86 | 0.71 | No | No | No | No | Negative |
| Pembrolizumab (Genetical Recombination) | 91 | 0.44 | 0.36 | 0.54 | 0.44 | 0.36 | 0.54 | -1.16 | -1.45 | 0.45 | 0.38 | No | No | No | No | Negative |
| Voriconazole | 133 | 5.74 | 4.83 | 6.84 | 5.55 | 4.7 | 6.57 | 2.47 | 2.23 | 5.46 | 4.72 | Yes | Yes | Yes | Yes | Positive |
| Midazolam | 74 | 4.05 | 3.21 | 5.11 | 3.96 | 3.16 | 4.96 | 1.99 | 1.67 | 3.81 | 3.11 | Yes | Yes | Yes | Yes | Positive |
| Mirtazapine | 77 | 4.67 | 3.72 | 5.86 | 4.55 | 3.65 | 5.67 | 2.19 | 1.87 | 4.41 | 3.61 | Yes | Yes | Yes | Yes | Positive |
| Methotrexate | 134 | 0.5 | 0.42 | 0.59 | 0.5 | 0.42 | 0.59 | -0.98 | -1.23 | 0.51 | 0.44 | No | No | No | No | Negative |
| Metronidazole | 123 | 13.67 | 11.35 | 16.46 | 12.53 | 10.57 | 14.84 | 3.64 | 3.4 | 12.27 | 10.56 | Yes | Yes | Yes | Yes | Positive |
| Morphine Hydrochloride Hydrate | 86 | 12.1 | 9.7 | 15.1 | 11.21 | 9.14 | 13.73 | 3.49 | 3.2 | 10.97 | 9.15 | Yes | Yes | Yes | Yes | Positive |
| Lamotrigine | 147 | 2.54 | 2.16 | 2.99 | 2.51 | 2.14 | 2.95 | 1.32 | 1.09 | 2.45 | 2.14 | Yes | Yes | Yes | Yes | Positive |
| Rivastigmine | 80 | 5.55 | 4.44 | 6.95 | 5.38 | 4.33 | 6.67 | 2.43 | 2.12 | 5.26 | 4.33 | Yes | Yes | Yes | Yes | Positive |
| Ribavirin | 100 | 0.56 | 0.46 | 0.69 | 0.57 | 0.46 | 0.69 | -0.81 | -1.09 | 0.57 | 0.48 | No | No | No | No | Negative |
| Lenalidomide Hydrate | 182 | 1.44 | 1.25 | 1.67 | 1.44 | 1.24 | 1.66 | 0.52 | 0.32 | 1.42 | 1.26 | No | No | Yes | No | Negative |
| Levetiracetam | 82 | 2.06 | 1.66 | 2.57 | 2.05 | 1.65 | 2.54 | 1.04 | 0.73 | 1.99 | 1.65 | Yes | Yes | Yes | No | Negative |
| Loxoprofen Sodium Hydrate | 111 | 1.61 | 1.33 | 1.94 | 1.6 | 1.33 | 1.93 | 0.68 | 0.41 | 1.57 | 1.34 | No | No | Yes | No | Negative |
| Recombinant Adsorbed Bivalent Human Papillomavirus VLP Vaccine (Insect Cell) | 64 | 2.91 | 2.27 | 3.73 | 2.87 | 2.25 | 3.66 | 1.53 | 1.18 | 2.74 | 2.21 | Yes | Yes | Yes | Yes | Positive |
| **ATC L1** |  |  |  |  |  |  |  |  |  |  |  |  |  |  |  |  |
| ALIMENTARY TRACT AND METABOLISM | 1153 | 1.29 | 1.21 | 1.37 | 1.29 | 1.21 | 1.37 | 0.34 | 0.26 | 1.26 | 1.21 | No | No | Yes | No | Negative |
| BLOOD AND BLOOD FORMING ORGANS | 93 | 0.23 | 0.18 | 0.28 | 0.23 | 0.19 | 0.28 | -2.09 | -2.39 | 0.23 | 0.2 | No | No | No | No | Negative |
| CARDIOVASCULAR SYSTEM | 276 | 0.67 | 0.6 | 0.76 | 0.67 | 0.6 | 0.76 | -0.56 | -0.72 | 0.68 | 0.62 | No | No | No | No | Negative |
| DERMATOLOGICALS | 483 | 1.64 | 1.5 | 1.8 | 1.63 | 1.49 | 1.79 | 0.69 | 0.56 | 1.61 | 1.49 | No | No | Yes | No | Negative |
| ANTIINFECTIVES FOR SYSTEMIC USE | 658 | 0.91 | 0.84 | 0.98 | 0.91 | 0.84 | 0.98 | -0.13 | -0.24 | 0.91 | 0.85 | No | No | No | No | Negative |
| ANTINEOPLASTIC AND IMMUNOMODULATING AGENTS | 1342 | 0.44 | 0.42 | 0.47 | 0.44 | 0.42 | 0.47 | -1.02 | -1.09 | 0.49 | 0.47 | No | No | No | No | Negative |
| MUSCULO-SKELETAL SYSTEM | 69 | 0.51 | 0.4 | 0.65 | 0.51 | 0.41 | 0.65 | -0.95 | -1.28 | 0.52 | 0.42 | No | No | No | No | Negative |
| NERVOUS SYSTEM | 2756 | 3.11 | 2.98 | 3.24 | 3.07 | 2.95 | 3.2 | 1.42 | 1.37 | 2.68 | 2.59 | Yes | Yes | Yes | Yes | Positive |
| RESPIRATORY SYSTEM | 167 | 3.51 | 3.01 | 4.09 | 3.45 | 2.96 | 4.01 | 1.78 | 1.56 | 3.36 | 2.95 | Yes | Yes | Yes | Yes | Positive |
| VARIOUS | 174 | 1.08 | 0.93 | 1.25 | 1.08 | 0.93 | 1.25 | 0.11 | -0.1 | 1.07 | 0.95 | No | No | No | No | Negative |
| **ATC L2** |  |  |  |  |  |  |  |  |  |  |  |  |  |  |  |  |
| STOMATOLOGICAL PREPARATIONS | 591 | 1.22 | 1.13 | 1.33 | 1.22 | 1.12 | 1.33 | 0.28 | 0.16 | 1.21 | 1.13 | No | No | Yes | No | Negative |
| DRUGS FOR CONSTIPATION | 280 | 5.12 | 4.54 | 5.77 | 4.97 | 4.43 | 5.58 | 2.29 | 2.13 | 4.87 | 4.41 | Yes | Yes | Yes | Yes | Positive |
| OTHER DERMATOLOGICAL PREPARATIONS | 228 | 1.02 | 0.9 | 1.16 | 1.02 | 0.9 | 1.16 | 0.03 | -0.15 | 1.02 | 0.91 | No | No | No | No | Negative |
| ANTIVIRALS FOR SYSTEMIC USE | 263 | 0.74 | 0.66 | 0.84 | 0.74 | 0.66 | 0.84 | -0.42 | -0.59 | 0.75 | 0.68 | No | No | No | No | Negative |
| ANTINEOPLASTIC AGENTS | 964 | 0.39 | 0.37 | 0.42 | 0.4 | 0.37 | 0.42 | -1.2 | -1.29 | 0.44 | 0.41 | No | No | No | No | Negative |
| IMMUNOSUPPRESSANTS | 331 | 0.89 | 0.8 | 0.99 | 0.89 | 0.8 | 0.99 | -0.16 | -0.32 | 0.89 | 0.81 | No | No | No | No | Negative |
| ANALGESICS | 383 | 5 | 4.51 | 5.54 | 4.86 | 4.4 | 5.37 | 2.25 | 2.11 | 4.74 | 4.35 | Yes | Yes | Yes | Yes | Positive |
| ANTIEPILEPTICS | 420 | 1.74 | 1.58 | 1.92 | 1.73 | 1.57 | 1.9 | 0.77 | 0.64 | 1.7 | 1.57 | No | No | Yes | No | Negative |
| PSYCHOLEPTICS | 1304 | 2.69 | 2.54 | 2.85 | 2.66 | 2.52 | 2.82 | 1.33 | 1.26 | 2.51 | 2.4 | Yes | Yes | Yes | Yes | Positive |
| PSYCHOANALEPTICS | 228 | 3.8 | 3.33 | 4.33 | 3.72 | 3.27 | 4.23 | 1.88 | 1.7 | 3.64 | 3.25 | Yes | Yes | Yes | Yes | Positive |

Supplementary Table 4. Disproportionality signals for drug-associated delirium in CVAR: Top 50 drugs and top 10 ATC classifications at Level 1 (L1) and Level 2 (L2)

| **drugs** | **cases** | **ROR** | **ROR_lower** | **ROR_upper** | **PRR** | **PRR_lower** | **PRR_upper** | **IC** | **IC_lower** | **EBGM** | **EBGM_lower** | **ROR_positive** | **PRR_positive** | **IC_positive** | **EBGM_positive** | **overall_positive** |
| --- | --- | --- | --- | --- | --- | --- | --- | --- | --- | --- | --- | --- | --- | --- | --- | --- |
| METHOTREXATE | 865 | 1.38 | 1.29 | 1.48 | 1.38 | 1.29 | 1.47 | 0.43 | 0.34 | 1.35 | 1.28 | No | No | Yes | No | Negative |
| ACETAMINOPHEN | 856 | 7.65 | 7.12 | 8.23 | 7.13 | 6.67 | 7.62 | 2.74 | 2.66 | 6.69 | 6.32 | Yes | Yes | Yes | Yes | Positive |
| INFLIXIMAB | 784 | 0.6 | 0.56 | 0.65 | 0.61 | 0.57 | 0.65 | -0.66 | -0.76 | 0.63 | 0.6 | No | No | No | No | Negative |
| ADALIMUMAB | 730 | 0.78 | 0.73 | 0.84 | 0.78 | 0.73 | 0.84 | -0.33 | -0.43 | 0.8 | 0.75 | No | No | No | No | Negative |
| ETANERCEPT | 696 | 2.04 | 1.89 | 2.2 | 2.01 | 1.87 | 2.17 | 0.97 | 0.87 | 1.95 | 1.83 | Yes | Yes | Yes | No | Negative |
| RITUXIMAB | 684 | 1.78 | 1.65 | 1.93 | 1.77 | 1.64 | 1.91 | 0.79 | 0.68 | 1.72 | 1.62 | No | No | Yes | No | Negative |
| TOCILIZUMAB | 670 | 2.6 | 2.4 | 2.81 | 2.55 | 2.36 | 2.75 | 1.3 | 1.2 | 2.46 | 2.3 | Yes | Yes | Yes | Yes | Positive |
| ABATACEPT | 658 | 2.38 | 2.2 | 2.58 | 2.35 | 2.17 | 2.53 | 1.19 | 1.08 | 2.26 | 2.12 | Yes | Yes | Yes | Yes | Positive |
| PREDNISONE | 648 | 2.81 | 2.59 | 3.04 | 2.75 | 2.54 | 2.97 | 1.41 | 1.3 | 2.64 | 2.48 | Yes | Yes | Yes | Yes | Positive |
| OXYCODONE | 645 | 8.32 | 7.66 | 9.04 | 7.69 | 7.12 | 8.29 | 2.88 | 2.77 | 7.32 | 6.86 | Yes | Yes | Yes | Yes | Positive |
| LEFLUNOMIDE | 642 | 3.28 | 3.02 | 3.55 | 3.2 | 2.96 | 3.45 | 1.62 | 1.52 | 3.07 | 2.87 | Yes | Yes | Yes | Yes | Positive |
| CERTOLIZUMAB PEGOL | 587 | 4.26 | 3.91 | 4.64 | 4.11 | 3.79 | 4.45 | 1.99 | 1.87 | 3.94 | 3.68 | Yes | Yes | Yes | Yes | Positive |
| GOLIMUMAB | 576 | 4.33 | 3.97 | 4.71 | 4.17 | 3.84 | 4.52 | 2.01 | 1.9 | 4 | 3.73 | Yes | Yes | Yes | Yes | Positive |
| CLOZAPINE | 568 | 1.91 | 1.75 | 2.08 | 1.89 | 1.74 | 2.05 | 0.89 | 0.77 | 1.84 | 1.72 | No | No | Yes | No | Negative |
| CHLORHEXIDINE | 537 | 55.59 | 49.81 | 62.04 | 34.31 | 32.04 | 36.75 | 5.04 | 4.95 | 32.63 | 30.38 | Yes | Yes | Yes | Yes | Positive |
| HYDROXYCHLOROQUINE | 514 | 2.7 | 2.47 | 2.95 | 2.64 | 2.42 | 2.89 | 1.37 | 1.25 | 2.56 | 2.38 | Yes | Yes | Yes | Yes | Positive |
| DICLOFENAC | 504 | 5.89 | 5.38 | 6.46 | 5.58 | 5.12 | 6.08 | 2.43 | 2.31 | 5.38 | 5 | Yes | Yes | Yes | Yes | Positive |
| TOFACITINIB | 495 | 2.17 | 1.98 | 2.38 | 2.14 | 1.96 | 2.34 | 1.07 | 0.95 | 2.08 | 1.93 | Yes | Yes | Yes | Yes | Positive |
| ALENDRONATE | 475 | 12.78 | 11.59 | 14.1 | 11.27 | 10.34 | 12.27 | 3.44 | 3.32 | 10.84 | 10.04 | Yes | Yes | Yes | Yes | Positive |
| SULFAMETHOXAZOLE | 467 | 16.71 | 15.11 | 18.49 | 14.17 | 13.01 | 15.42 | 3.77 | 3.66 | 13.62 | 12.61 | Yes | Yes | Yes | Yes | Positive |
| DESOXIMETASONE | 464 | 21.91 | 19.76 | 24.3 | 17.68 | 16.27 | 19.22 | 4.09 | 3.98 | 16.98 | 15.72 | Yes | Yes | Yes | Yes | Positive |
| SULFASALAZINE | 458 | 2.69 | 2.45 | 2.96 | 2.64 | 2.41 | 2.89 | 1.37 | 1.24 | 2.56 | 2.37 | Yes | Yes | Yes | Yes | Positive |
| FOLIC ACID | 441 | 13.11 | 11.84 | 14.51 | 11.51 | 10.53 | 12.58 | 3.48 | 3.36 | 11.1 | 10.25 | Yes | Yes | Yes | Yes | Positive |
| CETIRIZINE | 411 | 19.71 | 17.68 | 21.98 | 16.22 | 14.84 | 17.74 | 3.98 | 3.85 | 15.64 | 14.41 | Yes | Yes | Yes | Yes | Positive |
| SECUKINUMAB | 411 | 1.92 | 1.74 | 2.12 | 1.9 | 1.73 | 2.1 | 0.91 | 0.77 | 1.86 | 1.72 | No | No | Yes | No | Negative |
| PSEUDOEPHEDRINE | 388 | 35.22 | 31.25 | 39.69 | 25.27 | 23.2 | 27.53 | 4.62 | 4.5 | 24.33 | 22.37 | Yes | Yes | Yes | Yes | Positive |
| CORTISONE | 365 | 23.55 | 20.95 | 26.48 | 18.69 | 17.04 | 20.51 | 4.18 | 4.06 | 18.06 | 16.55 | Yes | Yes | Yes | Yes | Positive |
| OCTREOTIDE | 351 | 4.26 | 3.82 | 4.75 | 4.1 | 3.7 | 4.55 | 2.01 | 1.86 | 3.99 | 3.65 | Yes | Yes | Yes | Yes | Positive |
| USTEKINUMAB | 310 | 2.24 | 1.99 | 2.51 | 2.2 | 1.97 | 2.46 | 1.12 | 0.97 | 2.16 | 1.96 | Yes | Yes | Yes | Yes | Positive |
| PHTHALYLSULFATHIAZOLE | 306 | 26.3 | 23.11 | 29.94 | 20.34 | 18.41 | 22.47 | 4.31 | 4.17 | 19.71 | 17.92 | Yes | Yes | Yes | Yes | Positive |
| CALCIUM CARBONATE | 302 | 13.92 | 12.31 | 15.75 | 12.11 | 10.89 | 13.47 | 3.57 | 3.42 | 11.78 | 10.71 | Yes | Yes | Yes | Yes | Positive |
| PREGABALIN | 296 | 5.28 | 4.69 | 5.95 | 5.03 | 4.5 | 5.63 | 2.3 | 2.15 | 4.91 | 4.45 | Yes | Yes | Yes | Yes | Positive |
| QUETIAPINE | 285 | 5.39 | 4.78 | 6.09 | 5.13 | 4.58 | 5.75 | 2.33 | 2.17 | 5.01 | 4.53 | Yes | Yes | Yes | Yes | Positive |
| HYDROCORTISONE | 283 | 10.78 | 9.51 | 12.21 | 9.68 | 8.66 | 10.82 | 3.25 | 3.09 | 9.44 | 8.55 | Yes | Yes | Yes | Yes | Positive |
| CELECOXIB | 272 | 3.89 | 3.44 | 4.4 | 3.76 | 3.34 | 4.23 | 1.89 | 1.72 | 3.66 | 3.31 | Yes | Yes | Yes | Yes | Positive |
| MORPHINE | 270 | 4.01 | 3.54 | 4.54 | 3.87 | 3.44 | 4.36 | 1.93 | 1.77 | 3.77 | 3.41 | Yes | Yes | Yes | Yes | Positive |
| NAPROXEN | 269 | 5.23 | 4.62 | 5.92 | 4.98 | 4.43 | 5.6 | 2.29 | 2.13 | 4.87 | 4.39 | Yes | Yes | Yes | Yes | Positive |
| METHYLPREDNISOLONE | 234 | 5.88 | 5.14 | 6.72 | 5.56 | 4.91 | 6.3 | 2.45 | 2.28 | 5.44 | 4.88 | Yes | Yes | Yes | Yes | Positive |
| APREMILAST | 215 | 7.17 | 6.23 | 8.25 | 6.69 | 5.88 | 7.62 | 2.72 | 2.54 | 6.56 | 5.85 | Yes | Yes | Yes | Yes | Positive |
| PANTOPRAZOLE | 211 | 3.33 | 2.9 | 3.83 | 3.25 | 2.84 | 3.71 | 1.68 | 1.5 | 3.16 | 2.81 | Yes | Yes | Yes | Yes | Positive |
| RAMIPRIL | 210 | 6.75 | 5.86 | 7.78 | 6.33 | 5.55 | 7.21 | 2.64 | 2.46 | 6.2 | 5.53 | Yes | Yes | Yes | Yes | Positive |
| HYDROMORPHONE | 210 | 3.43 | 2.98 | 3.94 | 3.33 | 2.91 | 3.81 | 1.72 | 1.53 | 3.24 | 2.89 | Yes | Yes | Yes | Yes | Positive |
| METFORMIN | 205 | 5.18 | 4.5 | 5.97 | 4.94 | 4.32 | 5.65 | 2.29 | 2.1 | 4.84 | 4.3 | Yes | Yes | Yes | Yes | Positive |
| CANDESARTAN | 193 | 4.13 | 3.57 | 4.77 | 3.98 | 3.46 | 4.57 | 1.98 | 1.78 | 3.88 | 3.43 | Yes | Yes | Yes | Yes | Positive |
| CHOLECALCIFEROL | 192 | 25.74 | 21.87 | 30.28 | 19.96 | 17.62 | 22.63 | 4.3 | 4.12 | 19.44 | 17.23 | Yes | Yes | Yes | Yes | Positive |
| SARILUMAB | 190 | 15.93 | 13.63 | 18.62 | 13.57 | 11.89 | 15.47 | 3.74 | 3.56 | 13.26 | 11.74 | Yes | Yes | Yes | Yes | Positive |
| FLUMETHASONE | 189 | 60.79 | 50.38 | 73.34 | 35.79 | 32.05 | 39.96 | 5.14 | 4.99 | 34.55 | 30.61 | Yes | Yes | Yes | Yes | Positive |
| VEDOLIZUMAB | 183 | 0.56 | 0.48 | 0.64 | 0.56 | 0.48 | 0.65 | -0.82 | -1.03 | 0.57 | 0.5 | No | No | No | No | Negative |
| ASPIRIN | 175 | 3.17 | 2.72 | 3.69 | 3.09 | 2.67 | 3.58 | 1.62 | 1.41 | 3.01 | 2.65 | Yes | Yes | Yes | Yes | Positive |
| ASCORBIC ACID | 171 | 16.88 | 14.31 | 19.91 | 14.24 | 12.4 | 16.34 | 3.82 | 3.62 | 13.91 | 12.24 | Yes | Yes | Yes | Yes | Positive |
| **ATC L1** |  |  |  |  |  |  |  |  |  |  |  |  |  |  |  |  |
| A ALIMENTARY TRACT AND METABOLISM | 1719 | 1.66 | 1.58 | 1.75 | 1.65 | 1.57 | 1.74 | 0.64 | 0.58 | 1.56 | 1.5 | No | No | Yes | No | Negative |
| B BLOOD AND BLOOD FORMING ORGANS | 774 | 2.16 | 2.01 | 2.33 | 2.13 | 1.99 | 2.29 | 1.05 | 0.95 | 2.06 | 1.94 | Yes | Yes | Yes | Yes | Positive |
| C CARDIOVASCULAR SYSTEM | 1187 | 1.68 | 1.58 | 1.78 | 1.67 | 1.57 | 1.77 | 0.68 | 0.6 | 1.6 | 1.53 | No | No | Yes | No | Negative |
| D DERMATOLOGICALS | 1000 | 2.26 | 2.12 | 2.42 | 2.23 | 2.09 | 2.38 | 1.09 | 1.01 | 2.13 | 2.02 | Yes | Yes | Yes | Yes | Positive |
| H SYSTEMIC HORMONAL PREPARATIONS, EXCL. SEX HORMONES AND INSULINS | 905 | 4.55 | 4.24 | 4.88 | 4.38 | 4.1 | 4.67 | 2.05 | 1.96 | 4.12 | 3.89 | Yes | Yes | Yes | Yes | Positive |
| J ANTIINFECTIVES FOR SYSTEMIC USE | 1329 | 3.26 | 3.08 | 3.46 | 3.18 | 3.01 | 3.37 | 1.56 | 1.49 | 2.94 | 2.81 | Yes | Yes | Yes | Yes | Positive |
| L ANTINEOPLASTIC AND IMMUNOMODULATING AGENTS | 4340 | 0.52 | 0.5 | 0.54 | 0.53 | 0.51 | 0.55 | -0.52 | -0.56 | 0.7 | 0.68 | No | No | No | No | Negative |
| N NERVOUS SYSTEM | 4222 | 3.33 | 3.2 | 3.45 | 3.26 | 3.14 | 3.38 | 1.31 | 1.28 | 2.48 | 2.42 | Yes | Yes | Yes | Yes | Positive |
| P ANTIPARASITIC PRODUCTS, INSECTICIDES AND REPELLENTS | 558 | 2.72 | 2.5 | 2.97 | 2.67 | 2.45 | 2.9 | 1.37 | 1.26 | 2.58 | 2.4 | Yes | Yes | Yes | Yes | Positive |
| R RESPIRATORY SYSTEM | 949 | 1.7 | 1.59 | 1.82 | 1.69 | 1.58 | 1.8 | 0.71 | 0.62 | 1.63 | 1.55 | No | No | Yes | No | Negative |
| **ATC l2** |  |  |  |  |  |  |  |  |  |  |  |  |  |  |  |  |
| A01 STOMATOLOGICAL PREPARATIONS | 884 | 5.03 | 4.69 | 5.4 | 4.81 | 4.5 | 5.15 | 2.18 | 2.09 | 4.53 | 4.28 | Yes | Yes | Yes | Yes | Positive |
| A07 ANTIDIARRHEALS, INTESTINAL ANTIINFLAMMATORY/ANTIINFECTIVE AGENTS | 761 | 1.53 | 1.42 | 1.65 | 1.52 | 1.41 | 1.63 | 0.57 | 0.47 | 1.48 | 1.4 | No | No | Yes | No | Negative |
| D07 CORTICOSTEROIDS, DERMATOLOGICAL PREPARATIONS | 595 | 5.22 | 4.8 | 5.69 | 4.98 | 4.6 | 5.4 | 2.26 | 2.15 | 4.78 | 4.46 | Yes | Yes | Yes | Yes | Positive |
| D11 OTHER DERMATOLOGICAL PREPARATIONS | 613 | 3.19 | 2.93 | 3.46 | 3.11 | 2.87 | 3.37 | 1.59 | 1.48 | 2.99 | 2.79 | Yes | Yes | Yes | Yes | Positive |
| J01 ANTIBACTERIALS FOR SYSTEMIC USE | 957 | 3.28 | 3.07 | 3.51 | 3.2 | 3 | 3.41 | 1.6 | 1.51 | 3.02 | 2.86 | Yes | Yes | Yes | Yes | Positive |
| L01 ANTINEOPLASTIC AGENTS | 2187 | 1.14 | 1.09 | 1.2 | 1.14 | 1.09 | 1.2 | 0.16 | 0.11 | 1.12 | 1.08 | No | No | Yes | No | Negative |
| L04 IMMUNOSUPPRESSANTS | 2738 | 0.49 | 0.47 | 0.51 | 0.49 | 0.47 | 0.51 | -0.73 | -0.78 | 0.6 | 0.59 | No | No | No | No | Negative |
| N02 ANALGESICS | 1466 | 3.59 | 3.4 | 3.8 | 3.49 | 3.31 | 3.69 | 1.68 | 1.61 | 3.19 | 3.05 | Yes | Yes | Yes | Yes | Positive |
| N05 PSYCHOLEPTICS | 1419 | 2.77 | 2.62 | 2.93 | 2.72 | 2.57 | 2.87 | 1.33 | 1.26 | 2.51 | 2.41 | Yes | Yes | Yes | Yes | Positive |
| N06 PSYCHOANALEPTICS | 1203 | 3.31 | 3.11 | 3.51 | 3.22 | 3.04 | 3.42 | 1.59 | 1.51 | 3 | 2.86 | Yes | Yes | Yes | Yes | Positive |

Supplementary Table 5. Time-to-Onset Summary by ATC Level 1 Class (FAERS and JADER)

| **Database** | **ATC_L1** | **Name_L1** | **N** | **Median_TTO_days** | **Q1** | **Q3** | **IQR** | **Median_IQR_str** | **Pct_le_7d** | **Pct_le_14d** | **Signal_strength** | **N_flag** |
| --- | --- | --- | --- | --- | --- | --- | --- | --- | --- | --- | --- | --- |
| FAERS | J | ANTIINFECTIVES FOR SYSTEMIC USE | 3007 | 3 | 0 | 10 | 10 | 3.0 (0.0-10.0) | 69.1 | 81.6 | Strong |  |
| FAERS | V | VARIOUS | 526 | 0 | 0 | 1 | 1 | 0.0 (0.0-1.0) | 89.2 | 90.9 | Strong |  |
| FAERS | L | ANTINEOPLASTIC AND IMMUNOMODULATING AGENTS | 10039 | 14 | 2 | 63 | 61 | 14.0 (2.0-63.0) | 40.3 | 50.7 | Moderate |  |
| FAERS | N | NERVOUS SYSTEM | 7337 | 2 | 0 | 20 | 20 | 2.0 (0.0-20.0) | 63.9 | 71.5 | Moderate |  |
| FAERS | A | ALIMENTARY TRACT AND METABOLISM | 2577 | 2 | 0 | 17 | 17 | 2.0 (0.0-17.0) | 66.2 | 73.7 | Moderate |  |
| FAERS | C | CARDIOVASCULAR SYSTEM | 1584 | 8 | 0 | 50 | 50 | 8.0 (0.0-50.0) | 49.5 | 59 | Moderate |  |
| FAERS | B | BLOOD AND BLOOD FORMING ORGANS | 1195 | 2 | 0 | 49 | 49 | 2.0 (0.0-49.0) | 59.8 | 64.4 | Moderate |  |
| FAERS | D | DERMATOLOGICALS | 708 | 3 | 0 | 17 | 17 | 3.0 (0.0-17.0) | 63.4 | 72.5 | Moderate |  |
| FAERS | R | RESPIRATORY SYSTEM | 567 | 1 | 0 | 21 | 21 | 1.0 (0.0-21.0) | 69.1 | 73.7 | Moderate |  |
| FAERS | M | MUSCULO-SKELETAL SYSTEM | 498 | 2 | 0 | 15 | 15 | 2.0 (0.0-15.0) | 67.1 | 74.7 | Moderate |  |
| FAERS | H | SYSTEMIC HORMONAL PREPARATIONS, EXCL. SEX HORMONES AND INSULINS | 387 | 11 | 0 | 56.5 | 56.5 | 11.0 (0.0-56.5) | 46 | 54 | Moderate |  |
| FAERS | G | GENITO URINARY SYSTEM AND SEX HORMONES | 346 | 2 | 0 | 15 | 15 | 2.0 (0.0-15.0) | 65.6 | 73.1 | Moderate |  |
| FAERS | S | SENSORY ORGANS | 76 | 2 | 0 | 23.5 | 23.5 | 2.0 (0.0-23.5) | 64.5 | 71.1 | Moderate |  |
| FAERS | P | ANTIPARASITIC PRODUCTS, INSECTICIDES AND REPELLENTS | 34 | 2 | 0 | 17.8 | 17.8 | 2.0 (0.0-17.8) | 67.6 | 73.5 | Moderate |  |
| JADER | J | ANTIINFECTIVES FOR SYSTEMIC USE | 375 | 3 | 1 | 10 | 9 | 3.0 (1.0-10.0) | 72.3 | 79.5 | Strong |  |
| JADER | D | DERMATOLOGICALS | 165 | 3 | 1 | 8 | 7 | 3.0 (1.0-8.0) | 73.3 | 80 | Strong |  |
| JADER | V | VARIOUS | 118 | 0 | 0 | 2 | 2 | 0.0 (0.0-2.0) | 99.2 | 100 | Strong |  |
| JADER | R | RESPIRATORY SYSTEM | 38 | 2 | 0 | 5.8 | 5.8 | 2.0 (0.0-5.8) | 78.9 | 78.9 | Strong |  |
| JADER | S | SENSORY ORGANS | 3 | 5 | 3.5 | 9 | 5.5 | 5.0 (3.5-9.0) | 66.7 | 100 | Strong | N<5 |
| JADER | N | NERVOUS SYSTEM | 874 | 10 | 2 | 40 | 38 | 10.0 (2.0-40.0) | 46.7 | 57.1 | Moderate |  |
| JADER | L | ANTINEOPLASTIC AND IMMUNOMODULATING AGENTS | 605 | 12 | 3 | 43 | 40 | 12.0 (3.0-43.0) | 43.6 | 56.4 | Moderate |  |
| JADER | A | ALIMENTARY TRACT AND METABOLISM | 361 | 12 | 2 | 40 | 38 | 12.0 (2.0-40.0) | 43.2 | 56 | Moderate |  |
| JADER | C | CARDIOVASCULAR SYSTEM | 75 | 9 | 3 | 39.5 | 36.5 | 9.0 (3.0-39.5) | 48 | 53.3 | Moderate |  |
| JADER | B | BLOOD AND BLOOD FORMING ORGANS | 37 | 10 | 1 | 25 | 24 | 10.0 (1.0-25.0) | 48.6 | 64.9 | Moderate |  |
| JADER | M | MUSCULO-SKELETAL SYSTEM | 27 | 3 | 1 | 30 | 29 | 3.0 (1.0-30.0) | 63 | 63 | Moderate |  |
| JADER | P | ANTIPARASITIC PRODUCTS, INSECTICIDES AND REPELLENTS | 4 | 8.5 | 3.2 | 13 | 9.8 | 8.5 (3.2-13.0) | 50 | 100 | Moderate | N<5 |
| JADER | G | GENITO URINARY SYSTEM AND SEX HORMONES | 14 | 28 | 17.2 | 67.5 | 50.2 | 28.0 (17.2-67.5) | 14.3 | 21.4 | Weak |  |

Supplementary Table 6. Time-to-Onset Summary by ATC Level 2 Class (FAERS and JADER)

| **Database** | **ATC_L2** | **Name_L2** | **N** | **Median_TTO_days** | **Q1** | **Q3** | **IQR** | **Median_IQR_str** | **Pct_le_7d** | **Pct_le_14d** | **Signal_strength** | **N_flag** |
| --- | --- | --- | --- | --- | --- | --- | --- | --- | --- | --- | --- | --- |
| FAERS | L01 | ANTINEOPLASTIC AGENTS | 6606 | 12 | 2 | 49 | 47 | 12.0 (2.0-49.0) | 42.7 | 54.5 | Moderate |  |
| FAERS | N05 | PSYCHOLEPTICS | 2924 | 1 | 0 | 19 | 19 | 1.0 (0.0-19.0) | 65.5 | 73 | Moderate |  |
| FAERS | L04 | IMMUNOSUPPRESSANTS | 2692 | 22 | 2 | 103 | 101 | 22.0 (2.0-103.0) | 35.6 | 43.8 | Moderate |  |
| FAERS | J01 | ANTIBACTERIALS FOR SYSTEMIC USE | 1836 | 3 | 0 | 8 | 8 | 3.0 (0.0-8.0) | 74 | 87.4 | Strong |  |
| FAERS | N02 | ANALGESICS | 1450 | 2 | 0 | 17 | 17 | 2.0 (0.0-17.0) | 66.5 | 73.4 | Moderate |  |
| FAERS | N06 | PSYCHOANALEPTICS | 1053 | 2 | 0 | 21 | 21 | 2.0 (0.0-21.0) | 61.6 | 69.6 | Moderate |  |
| FAERS | N03 | ANTIEPILEPTICS | 959 | 3 | 0 | 22 | 22 | 3.0 (0.0-22.0) | 59.5 | 68.4 | Moderate |  |
| FAERS | J05 | ANTIVIRALS FOR SYSTEMIC USE | 803 | 6 | 1 | 29 | 28 | 6.0 (1.0-29.0) | 52.9 | 65.6 | Moderate |  |
| FAERS | B01 | ANTITHROMBOTIC AGENTS | 651 | 16 | 1 | 113 | 112 | 16.0 (1.0-113.0) | 43.2 | 49 | Moderate |  |
| FAERS | A10 | DRUGS USED IN DIABETES | 619 | 7 | 0 | 62.5 | 62.5 | 7.0 (0.0-62.5) | 52 | 59 | Moderate |  |
| FAERS | A01 | STOMATOLOGICAL PREPARATIONS | 559 | 2 | 0 | 9.5 | 9.5 | 2.0 (0.0-9.5) | 71.4 | 82.8 | Strong |  |
| FAERS | L02 | ENDOCRINE THERAPY | 469 | 30 | 6 | 88 | 82 | 30.0 (6.0-88.0) | 27.9 | 34.8 | Weak |  |
| FAERS | D11 | OTHER DERMATOLOGICAL PREPARATIONS | 398 | 5 | 0 | 39 | 39 | 5.0 (0.0-39.0) | 52.8 | 60.6 | Moderate |  |
| FAERS | B05 | BLOOD SUBSTITUTES AND PERFUSION SOLUTIONS | 390 | 0 | 0 | 0 | 0 | 0.0 (0.0-0.0) | 95.6 | 96.2 | Strong |  |
| FAERS | N07 | OTHER NERVOUS SYSTEM DRUGS | 388 | 3 | 0 | 32.2 | 32.2 | 3.0 (0.0-32.2) | 60.6 | 64.7 | Moderate |  |
| FAERS | N01 | ANESTHETICS | 372 | 1 | 0 | 11.5 | 11.5 | 1.0 (0.0-11.5) | 69.9 | 79 | Strong |  |
| FAERS | C01 | CARDIAC THERAPY | 357 | 2 | 0 | 12 | 12 | 2.0 (0.0-12.0) | 68.6 | 79 | Strong |  |
| FAERS | C03 | DIURETICS | 346 | 10.5 | 1 | 54.8 | 53.8 | 10.5 (1.0-54.8) | 42.2 | 56.4 | Moderate |  |
| FAERS | C10 | LIPID MODIFYING AGENTS | 326 | 9 | 1 | 45.8 | 44.8 | 9.0 (1.0-45.8) | 46.6 | 59.8 | Moderate |  |
| FAERS | R03 | DRUGS FOR OBSTRUCTIVE AIRWAY DISEASES | 293 | 4 | 0 | 48 | 48 | 4.0 (0.0-48.0) | 53.9 | 56.7 | Moderate |  |
| FAERS | L03 | IMMUNOSTIMULANTS | 272 | 9 | 0 | 38.2 | 38.2 | 9.0 (0.0-38.2) | 47.4 | 57 | Moderate |  |
| FAERS | A06 | DRUGS FOR CONSTIPATION | 268 | 0 | 0 | 2 | 2 | 0.0 (0.0-2.0) | 85.1 | 85.8 | Strong |  |
| FAERS | A09 | DIGESTIVES, INCL. ENZYMES | 253 | 0 | 0 | 10 | 10 | 0.0 (0.0-10.0) | 73.5 | 77.9 | Strong |  |
| FAERS | A07 | ANTIDIARRHEALS, INTESTINAL ANTIINFLAMMATORY/ANTIINFECTIVE AGENTS | 247 | 6 | 1 | 20.5 | 19.5 | 6.0 (1.0-20.5) | 54.7 | 68.8 | Moderate |  |
| FAERS | M03 | MUSCLE RELAXANTS | 243 | 2 | 0 | 9.5 | 9.5 | 2.0 (0.0-9.5) | 72.4 | 79.8 | Strong |  |
| FAERS | C02 | ANTIHYPERTENSIVES | 238 | 35 | 4 | 128.2 | 124.2 | 35.0 (4.0-128.2) | 30.3 | 37 | Moderate |  |
| FAERS | V08 | CONTRAST MEDIA | 237 | 0 | 0 | 0 | 0 | 0.0 (0.0-0.0) | 100 | 100 | Strong |  |
| FAERS | A04 | ANTIEMETICS AND ANTINAUSEANTS | 229 | 1 | 0 | 3 | 3 | 1.0 (0.0-3.0) | 88.2 | 92.1 | Strong |  |
| FAERS | A02 | DRUGS FOR ACID RELATED DISORDERS | 225 | 3 | 0 | 16 | 16 | 3.0 (0.0-16.0) | 66.2 | 73.3 | Moderate |  |
| FAERS | C09 | AGENTS ACTING ON THE RENIN-ANGIOTENSIN SYSTEM | 210 | 17 | 1 | 127.5 | 126.5 | 17.0 (1.0-127.5) | 42.9 | 45.2 | Moderate |  |
| JADER | L01 | ANTINEOPLASTIC AGENTS | 495 | 9 | 2 | 42 | 40 | 9.0 (2.0-42.0) | 46.9 | 57.2 | Moderate |  |
| JADER | N05 | PSYCHOLEPTICS | 339 | 12 | 1 | 57 | 56 | 12.0 (1.0-57.0) | 44.2 | 52.8 | Moderate |  |
| JADER | A01 | STOMATOLOGICAL PREPARATIONS | 192 | 18.5 | 6 | 45 | 39 | 18.5 (6.0-45.0) | 31.2 | 45.8 | Moderate |  |
| JADER | J05 | ANTIVIRALS FOR SYSTEMIC USE | 171 | 6 | 2 | 40 | 38 | 6.0 (2.0-40.0) | 59.6 | 66.1 | Moderate |  |
| JADER | N03 | ANTIEPILEPTICS | 157 | 16 | 4 | 52 | 48 | 16.0 (4.0-52.0) | 35.7 | 49 | Moderate |  |
| JADER | N02 | ANALGESICS | 141 | 5 | 1 | 16 | 15 | 5.0 (1.0-16.0) | 61 | 73.8 | Moderate |  |
| JADER | J01 | ANTIBACTERIALS FOR SYSTEMIC USE | 107 | 2 | 1 | 3 | 2 | 2.0 (1.0-3.0) | 88.8 | 90.7 | Strong |  |
| JADER | V04 | DIAGNOSTIC AGENTS | 105 | 0 | 0 | 2 | 2 | 0.0 (0.0-2.0) | 100 | 100 | Strong |  |
| JADER | N06 | PSYCHOANALEPTICS | 100 | 16.5 | 3 | 54.8 | 51.8 | 16.5 (3.0-54.8) | 41 | 48 | Moderate |  |
| JADER | L04 | IMMUNOSUPPRESSANTS | 88 | 14.5 | 7.8 | 59.8 | 52 | 14.5 (7.8-59.8) | 25 | 50 | Moderate |  |
| JADER | J02 | ANTIMYCOTICS FOR SYSTEMIC USE | 84 | 2 | 1 | 3.2 | 2.2 | 2.0 (1.0-3.2) | 85.7 | 94 | Strong |  |
| JADER | A06 | DRUGS FOR CONSTIPATION | 83 | 3 | 0 | 20.5 | 20.5 | 3.0 (0.0-20.5) | 63.9 | 73.5 | Moderate |  |
| JADER | D06 | ANTIBIOTICS AND CHEMOTHERAPEUTICS FOR DERMATOLOGICAL USE | 74 | 2 | 1 | 3 | 2 | 2.0 (1.0-3.0) | 97.3 | 100 | Strong |  |
| JADER | D11 | OTHER DERMATOLOGICAL PREPARATIONS | 71 | 8 | 3 | 33 | 30 | 8.0 (3.0-33.0) | 46.5 | 56.3 | Moderate |  |
| JADER | N01 | ANESTHETICS | 68 | 5 | 1 | 14 | 13 | 5.0 (1.0-14.0) | 61.8 | 76.5 | Strong |  |
| JADER | A09 | DIGESTIVES, INCL. ENZYMES | 44 | 17.5 | 6.2 | 52.5 | 46.2 | 17.5 (6.2-52.5) | 29.5 | 47.7 | Moderate |  |
| JADER | N07 | OTHER NERVOUS SYSTEM DRUGS | 39 | 12 | 3.5 | 38 | 34.5 | 12.0 (3.5-38.0) | 48.7 | 59 | Moderate |  |
| JADER | R03 | DRUGS FOR OBSTRUCTIVE AIRWAY DISEASES | 32 | 3 | 0 | 10.2 | 10.2 | 3.0 (0.0-10.2) | 75 | 75 | Strong |  |
| JADER | N04 | ANTI-PARKINSON DRUGS | 30 | 8.5 | 2.8 | 53.5 | 50.8 | 8.5 (2.8-53.5) | 46.7 | 53.3 | Moderate |  |
| JADER | C03 | DIURETICS | 25 | 6 | 1 | 35 | 34 | 6.0 (1.0-35.0) | 64 | 68 | Moderate |  |
| JADER | A02 | DRUGS FOR ACID RELATED DISORDERS | 25 | 3 | 2 | 12 | 10 | 3.0 (2.0-12.0) | 72 | 76 | Strong |  |
| JADER | C05 | VASOPROTECTIVES | 23 | 15 | 6 | 54.5 | 48.5 | 15.0 (6.0-54.5) | 43.5 | 43.5 | Moderate |  |
| JADER | L02 | ENDOCRINE THERAPY | 18 | 10 | 6.2 | 22 | 15.8 | 10.0 (6.2-22.0) | 38.9 | 61.1 | Moderate |  |
| JADER | B05 | BLOOD SUBSTITUTES AND PERFUSION SOLUTIONS | 16 | 5 | 1 | 18.8 | 17.8 | 5.0 (1.0-18.8) | 68.8 | 68.8 | Moderate |  |
| JADER | C01 | CARDIAC THERAPY | 13 | 4 | 1 | 17 | 16 | 4.0 (1.0-17.0) | 61.5 | 69.2 | Moderate |  |
| JADER | J04 | ANTIMYCOBACTERIALS | 13 | 10 | 10 | 48 | 38 | 10.0 (10.0-48.0) | 15.4 | 69.2 | Moderate |  |
| JADER | M01 | ANTIINFLAMMATORY AND ANTIRHEUMATIC PRODUCTS | 12 | 13.5 | 1 | 30 | 29 | 13.5 (1.0-30.0) | 50 | 50 | Moderate |  |
| JADER | B01 | ANTITHROMBOTIC AGENTS | 11 | 10 | 1.5 | 19 | 17.5 | 10.0 (1.5-19.0) | 45.5 | 72.7 | Moderate |  |
| JADER | B03 | ANTIANEMIC PREPARATIONS | 9 | 21 | 14 | 30 | 16 | 21.0 (14.0-30.0) | 22.2 | 44.4 | Moderate | N<10 |
| JADER | D09 | MEDICATED DRESSINGS | 8 | 4.5 | 2 | 9.5 | 7.5 | 4.5 (2.0-9.5) | 62.5 | 87.5 | Strong | N<10 |

Supplementary Table 7. Time-to-Onset Summary by Individual Drug

| **Database** | **prod_ai** | **N** | **Median_TTO_days** | **Q1** | **Q3** | **IQR** | **Median_IQR_str** | **Pct_le_7d** | **Pct_le_14d** | **Signal_strength** |
| --- | --- | --- | --- | --- | --- | --- | --- | --- | --- | --- |
| FAERS | PIMAVANSERIN TARTRATE | 774 | 9 | 1 | 59 | 58 | 9.0 (1.0-59.0) | 48.3 | 57.4 | Moderate |
| FAERS | NIVOLUMAB | 529 | 33 | 10 | 105 | 95 | 33.0 (10.0-105.0) | 19.8 | 32.3 | Weak |
| FAERS | SERTRALINE HYDROCHLORIDE | 442 | 2 | 0 | 19 | 19 | 2.0 (0.0-19.0) | 62.7 | 72.9 | Moderate |
| FAERS | GABAPENTIN | 437 | 3 | 1 | 36 | 35 | 3.0 (1.0-36.0) | 60.4 | 65.7 | Moderate |
| FAERS | LENALIDOMIDE | 434 | 24 | 8 | 92.8 | 84.8 | 24.0 (8.0-92.8) | 24.2 | 37.1 | Weak |
| FAERS | AXICABTAGENE CILOLEUCEL | 420 | 2 | 1 | 5 | 4 | 2.0 (1.0-5.0) | 84.8 | 93.1 | Strong |
| FAERS | LEVOFLOXACIN | 358 | 2 | 0 | 5 | 5 | 2.0 (0.0-5.0) | 81.3 | 89.9 | Strong |
| FAERS | CIPROFLOXACIN | 330 | 2 | 0 | 9 | 9 | 2.0 (0.0-9.0) | 71.8 | 82.4 | Strong |
| FAERS | PREGABALIN | 327 | 2 | 0 | 14 | 14 | 2.0 (0.0-14.0) | 67.3 | 75.2 | Strong |
| FAERS | CILASTATIN SODIUM\IMIPENEM | 310 | 2 | 1 | 4 | 3 | 2.0 (1.0-4.0) | 90.3 | 99.4 | Strong |
| FAERS | SODIUM CITRATE | 294 | 0 | 0 | 0 | 0 | 0.0 (0.0-0.0) | 100 | 100 | Strong |
| FAERS | OLANZAPINE | 286 | 0 | 0 | 5 | 5 | 0.0 (0.0-5.0) | 78.7 | 85.3 | Strong |
| FAERS | QUETIAPINE | 284 | 0 | 0 | 6.2 | 6.2 | 0.0 (0.0-6.2) | 77.8 | 86.3 | Strong |
| FAERS | OXYCODONE HYDROCHLORIDE | 282 | 2 | 0 | 5.8 | 5.8 | 2.0 (0.0-5.8) | 78.7 | 86.5 | Strong |
| FAERS | ENZALUTAMIDE | 282 | 29.5 | 6 | 85 | 79 | 29.5 (6.0-85.0) | 28 | 35.5 | Weak |
| FAERS | CARBIDOPA\LEVODOPA | 271 | 57 | 6 | 187 | 181 | 57.0 (6.0-187.0) | 26.9 | 33.6 | Weak |
| FAERS | MOXIFLOXACIN HYDROCHLORIDE | 236 | 2 | 0 | 3 | 3 | 2.0 (0.0-3.0) | 94.1 | 98.3 | Strong |
| FAERS | TRAMADOL | 236 | 1 | 0 | 9 | 9 | 1.0 (0.0-9.0) | 74.2 | 82.6 | Strong |
| FAERS | FINGOLIMOD HYDROCHLORIDE | 231 | 7 | 0 | 77.5 | 77.5 | 7.0 (0.0-77.5) | 50.2 | 54.5 | Moderate |
| FAERS | ALPRAZOLAM | 225 | 0 | 0 | 0 | 0 | 0.0 (0.0-0.0) | 89.3 | 92.9 | Strong |
| JADER | Valaciclovir Hydrochloride | 444 | 2 | 1 | 3 | 2 | 2.0 (1.0-3.0) | 98 | 99.3 | Strong |
| JADER | SARS-CoV-2 RNA Vaccine | 168 | 1 | 0 | 4 | 4 | 1.0 (0.0-4.0) | 85.7 | 89.9 | Strong |
| JADER | Oxycodone Hydrochloride Hydrate | 145 | 5 | 2 | 20 | 18 | 5.0 (2.0-20.0) | 57.9 | 70.3 | Moderate |
| JADER | Pregabalin | 109 | 5 | 2 | 17 | 15 | 5.0 (2.0-17.0) | 56.9 | 70.6 | Moderate |
| JADER | Oseltamivir Phosphate | 105 | 0 | 0 | 2 | 2 | 0.0 (0.0-2.0) | 100 | 100 | Strong |
| JADER | Clozapine | 90 | 55 | 18.5 | 87.2 | 68.8 | 55.0 (18.5-87.2) | 8.9 | 16.7 | Weak |
| JADER | Zolpidem Tartrate | 80 | 3 | 0 | 19.2 | 19.2 | 3.0 (0.0-19.2) | 65 | 73.8 | Moderate |
| JADER | Fluorouracil | 78 | 26 | 2 | 110.8 | 108.8 | 26.0 (2.0-110.8) | 35.9 | 42.3 | Moderate |
| JADER | Aciclovir | 74 | 2 | 1 | 3 | 2 | 2.0 (1.0-3.0) | 97.3 | 100 | Strong |
| JADER | Voriconazole | 73 | 2 | 1 | 4 | 3 | 2.0 (1.0-4.0) | 84.9 | 94.5 | Strong |
| JADER | Nivolumab (Genetical Recombination) | 71 | 25 | 8 | 99 | 91 | 25.0 (8.0-99.0) | 22.5 | 39.4 | Weak |
| JADER | Dexamethasone | 70 | 13 | 3 | 30 | 27 | 13.0 (3.0-30.0) | 37.1 | 60 | Moderate |
| JADER | Tapentadol Hydrochloride | 67 | 4 | 1.5 | 9 | 7.5 | 4.0 (1.5-9.0) | 68.7 | 76.1 | Strong |
| JADER | Zanamivir Hydrate | 63 | 0 | 0 | 1 | 1 | 0.0 (0.0-1.0) | 100 | 100 | Strong |
| JADER | Fentanyl | 62 | 5 | 2 | 14 | 12 | 5.0 (2.0-14.0) | 59.7 | 75.8 | Moderate |
| JADER | Ribavirin | 61 | 40 | 7 | 83 | 76 | 40.0 (7.0-83.0) | 26.2 | 34.4 | Weak |
| JADER | Lamotrigine | 61 | 23 | 10 | 47 | 37 | 23.0 (10.0-47.0) | 23 | 42.6 | Weak |
| JADER | Prednisolone | 60 | 38.5 | 6 | 67 | 61 | 38.5 (6.0-67.0) | 30 | 38.3 | Moderate |
| JADER | Tacrolimus Hydrate | 58 | 15.5 | 4.2 | 42.8 | 38.5 | 15.5 (4.2-42.8) | 37.9 | 50 | Moderate |
| JADER | Ifosfamide | 56 | 4 | 2 | 10.2 | 8.2 | 4.0 (2.0-10.2) | 73.2 | 75 | Strong |

Supplementary Table 8. Trend Regression Complete Results (FAERS, JADER, and CVAR)

| Database | main_category | sub_category | group | slope | slope_lower | slope_upper | p_value | sig_level | fdr | sig_level_fdr |
| --- | --- | --- | --- | --- | --- | --- | --- | --- | --- | --- |
| FAERS | Overall Trend | Overall Trend | Overall | 540.51 | 408.9 | 672.11 | 3.98130561800057e-08 | *** | 7.7635459551011e-07 | *** |
| FAERS | Drug | ATC L1 | H SYSTEMIC HORMONAL PREPARATIONS, EXCL. SEX HORMONES AND INSULINS | -12.53 | -34.1 | 9.03 | 0.224360264102669 |  | 0.416669061904957 |  |
| FAERS | Drug | ATC L1 | C CARDIOVASCULAR SYSTEM | -16.82 | -61.96 | 28.32 | 0.425760860389986 |  | 0.664186942208378 |  |
| FAERS | Drug | ATC L1 | L ANTINEOPLASTIC AND IMMUNOMODULATING AGENTS | 141.22 | 38.82 | 243.62 | 0.0117863474353096 | * | 0.041787959088825 | * |
| FAERS | Drug | ATC L1 | V VARIOUS | -0.14 | -5.91 | 5.63 | 0.957995686962592 |  | 0.981576268715383 |  |
| FAERS | Drug | ATC L1 | B BLOOD AND BLOOD FORMING ORGANS | 10.95 | -6.35 | 28.26 | 0.188673417261624 |  | 0.387289199229548 |  |
| FAERS | Drug | ATC L1 | N NERVOUS SYSTEM | 89.29 | -33.17 | 211.74 | 0.135314226005953 |  | 0.32982842588951 |  |
| FAERS | Drug | ATC L1 | J ANTIINFECTIVES FOR SYSTEMIC USE | -0.25 | -23.94 | 23.44 | 0.981576268715383 |  | 0.981576268715383 |  |
| FAERS | Drug | ATC L1 | M MUSCULO-SKELETAL SYSTEM | 10.36 | -33.25 | 53.98 | 0.608039443132879 |  | 0.773089021361106 |  |
| FAERS | Drug | ATC L1 | A ALIMENTARY TRACT AND METABOLISM | 31.33 | -12.79 | 75.44 | 0.144715305351235 |  | 0.331993935805774 |  |
| FAERS | Drug | ATC L1 | D DERMATOLOGICALS | 40.27 | 31.31 | 49.24 | 1.57452102415158e-06 | *** | 8.77233142027307e-06 | *** |
| FAERS | Drug | ATC L2 | H01 PITUITARY AND HYPOTHALAMIC HORMONES AND ANALOGUES | -1.11 | -5.35 | 3.13 | 0.572142915375699 |  | 0.769433575850079 |  |
| FAERS | Drug | ATC L2 | C02 ANTIHYPERTENSIVES | -5.08 | -15.45 | 5.29 | 0.300302324049618 |  | 0.509208288605874 |  |
| FAERS | Drug | ATC L2 | L04 IMMUNOSUPPRESSANTS | 64.64 | 18.06 | 111.23 | 0.0114098454411916 | * | 0.041787959088825 | * |
| FAERS | Drug | ATC L2 | L01 ANTINEOPLASTIC AGENTS | 100.97 | 50.14 | 151.8 | 0.0012822324562199 | ** | 0.0055563406436199 | ** |
| FAERS | Drug | ATC L2 | V03 ALL OTHER THERAPEUTIC PRODUCTS | -0.09 | -2.46 | 2.29 | 0.936228495942244 |  | 0.981576268715383 |  |
| FAERS | Drug | ATC L2 | C03 DIURETICS | -2.1 | -14.08 | 9.88 | 0.704174594122447 |  | 0.858212786586732 |  |
| FAERS | Drug | ATC L2 | B01 ANTITHROMBOTIC AGENTS | -1.8 | -14.59 | 10.99 | 0.760674602944326 |  | 0.872538515142021 |  |
| FAERS | Drug | ATC L2 | N02 ANALGESICS | 9.3 | -25.77 | 44.36 | 0.567766814405511 |  | 0.769433575850079 |  |
| FAERS | Drug | ATC L2 | N03 ANTIEPILEPTICS | 8.01 | -16.73 | 32.75 | 0.487209965803807 |  | 0.73081494870571 |  |
| FAERS | Drug | ATC L2 | J05 ANTIVIRALS FOR SYSTEMIC USE | -9.34 | -22.01 | 3.33 | 0.131445250527171 |  | 0.32982842588951 |  |
| FAERS | Drug | Drug Name | ADALIMUMAB | 0.33 | -10.8 | 11.46 | 0.948824600115639 |  | 0.981576268715383 |  |
| FAERS | Drug | Drug Name | OXYCODONE HYDROCHLORIDE | 5.15 | -11.97 | 22.27 | 0.517817148903281 |  | 0.747958103971406 |  |
| FAERS | Drug | Drug Name | LENALIDOMIDE | -1.73 | -13.69 | 10.23 | 0.753679019936175 |  | 0.872538515142021 |  |
| FAERS | Drug | Drug Name | PREGABALIN | 1.01 | -11.58 | 13.6 | 0.862116250668962 |  | 0.960643822173986 |  |
| FAERS | Drug | Drug Name | BACLOFEN | 10.33 | -33.93 | 54.58 | 0.614506658004982 |  | 0.773089021361106 |  |
| FAERS | Drug | Drug Name | GABAPENTIN | 4.2 | -6.48 | 14.89 | 0.401287728857384 |  | 0.652092559393249 |  |
| FAERS | Drug | Drug Name | DULOXETINE HYDROCHLORIDE | -52.14 | -134.5 | 30.22 | 0.188679353470806 |  | 0.387289199229548 |  |
| FAERS | Drug | Drug Name | CARBIDOPA\LEVODOPA | 15.25 | 0.99 | 29.5 | 0.0383750059217984 | * | 0.106901802210724 |  |
| FAERS | Drug | Drug Name | METFORMIN HYDROCHLORIDE | 5.97 | -3.78 | 15.73 | 0.202503302173847 |  | 0.394881439239001 |  |
| FAERS | Drug | Drug Name | PIMAVANSERIN TARTRATE | 44.45 | 11.25 | 77.65 | 0.014944595965325 | * | 0.0485699368873062 | * |
| FAERS | Demographics | Age Group | <60 | 121.33 | 85.66 | 157 | 7.0618780686691e-07 | *** | 4.89735945444206e-06 | *** |
| FAERS | Demographics | Age Group | >=60 | 237.12 | 167.1 | 307.14 | 7.53439916068009e-07 | *** | 4.89735945444206e-06 | *** |
| FAERS | Demographics | Age Group |  | 182.05 | 131.05 | 233.05 | 3.46811804438664e-07 | *** | 3.38141509327697e-06 | *** |
| FAERS | Demographics | Gender | M | 189.73 | 129.86 | 249.59 | 1.93975512664583e-06 | *** | 9.45630624239844e-06 | *** |
| FAERS | Demographics | Gender | F | 257.98 | 191.46 | 324.51 | 9.81516219211684e-08 | *** | 1.27597108497519e-06 | *** |
| FAERS | Demographics | Gender | NS | 29.06 | 6.15 | 51.97 | 0.0199458481429773 | * | 0.059837544428932 |  |
| FAERS | Demographics | Gender |  | 92.32 | 72.52 | 112.11 | 7.740374569885291e-09 | *** | 3.01874608225526e-07 | *** |
| FAERS | Demographics | Gender | UNK | 0.29 | -0.27 | 0.85 | 0.290932282600837 |  | 0.509208288605874 |  |
| JADER | Overall Trend | Overall Trend | Overall | 10.89 | 4.39 | 17.39 | 0.0022772156973116 | ** | 0.0130801441151526 | * |
| JADER | Drug | ATC L1 | N NERVOUS SYSTEM | 1.72 | -3.37 | 6.8 | 0.489044127377137 |  | 0.640816442770041 |  |
| JADER | Drug | ATC L1 | G GENITO URINARY SYSTEM AND SEX HORMONES | -0.06 | -0.31 | 0.19 | 0.599391864112426 |  | 0.69020881322037 |  |
| JADER | Drug | ATC L1 | D DERMATOLOGICALS | -1.41 | -2.12 | -0.7 | 0.0005098839988719 | *** | 0.0052596263386056 | ** |
| JADER | Drug | ATC L1 | A ALIMENTARY TRACT AND METABOLISM | 0.6 | -1.47 | 2.66 | 0.554608527209653 |  | 0.664685215595534 |  |
| JADER | Drug | ATC L1 | V VARIOUS | -1.06 | -1.86 | -0.26 | 0.0122115968365455 | * | 0.0580050849735911 |  |
| JADER | Drug | ATC L1 | J ANTIINFECTIVES FOR SYSTEMIC USE | -0.65 | -1.81 | 0.5 | 0.253091350771761 |  | 0.422787322856027 |  |
| JADER | Drug | ATC L1 | R RESPIRATORY SYSTEM | -0.35 | -0.98 | 0.28 | 0.2558975901497 |  | 0.422787322856027 |  |
| JADER | Drug | ATC L1 | L ANTINEOPLASTIC AND IMMUNOMODULATING AGENTS | 0.14 | -1.61 | 1.89 | 0.867102131975334 |  | 0.890537324731424 |  |
| JADER | Drug | ATC L1 | C CARDIOVASCULAR SYSTEM | 0.09 | -0.38 | 0.57 | 0.692748733227093 |  | 0.752127196075129 |  |
| JADER | Drug | ATC L1 | B BLOOD AND BLOOD FORMING ORGANS | 0.19 | 0 | 0.38 | 0.0462044437388106 | * | 0.159615351097709 |  |
| JADER | Drug | ATC L2 | D06 ANTIBIOTICS AND CHEMOTHERAPEUTICS FOR DERMATOLOGICAL USE | -0.12 | -0.36 | 0.13 | 0.331726940706912 |  | 0.484831682571641 |  |
| JADER | Drug | ATC L2 | A02 DRUGS FOR ACID RELATED DISORDERS | 0 | -0.16 | 0.16 | 0.999999999999978 |  | 0.999999999999978 |  |
| JADER | Drug | ATC L2 | J01 ANTIBACTERIALS FOR SYSTEMIC USE | -0.34 | -0.79 | 0.12 | 0.138666065561306 |  | 0.301604989483797 |  |
| JADER | Drug | ATC L2 | J05 ANTIVIRALS FOR SYSTEMIC USE | -0.24 | -0.99 | 0.51 | 0.515079611103855 |  | 0.652434174064883 |  |
| JADER | Drug | ATC L2 | N05 PSYCHOLEPTICS | 1.44 | -0.62 | 3.49 | 0.160436071025797 |  | 0.316472430696811 |  |
| JADER | Drug | ATC L2 | L01 ANTINEOPLASTIC AGENTS | 1.02 | -0.31 | 2.35 | 0.124989580356315 |  | 0.296850253346247 |  |
| JADER | Drug | ATC L2 | A01 STOMATOLOGICAL PREPARATIONS | 0.42 | -1.33 | 2.17 | 0.623141818402896 |  | 0.696452620567943 |  |
| JADER | Drug | ATC L2 | L04 IMMUNOSUPPRESSANTS | -0.98 | -2.12 | 0.17 | 0.0916731618962883 |  | 0.267967704004535 |  |
| JADER | Drug | ATC L2 | N06 PSYCHOANALEPTICS | -0.15 | -0.67 | 0.37 | 0.559734918396239 |  | 0.664685215595534 |  |
| JADER | Drug | ATC L2 | N01 ANESTHETICS | -0.53 | -1.06 | 0.01 | 0.0536233593218131 |  | 0.169807304519075 |  |
| JADER | Drug | Drug Name | Aciclovir | -0.13 | -0.37 | 0.11 | 0.282825246376962 |  | 0.429894374492982 |  |
| JADER | Drug | Drug Name | Oxycodone Hydrochloride Hydrate | 0.7 | -0.59 | 1.99 | 0.268172902698848 |  | 0.424607095939842 |  |
| JADER | Drug | Drug Name | SARS-CoV-2 RNA Vaccine | -24.8 | -46.62 | -2.98 | 0.0363255940182564 | * | 0.138037257269374 |  |
| JADER | Drug | Drug Name | Zolpidem Tartrate | -0.28 | -0.7 | 0.13 | 0.166564437208848 |  | 0.316472430696811 |  |
| JADER | Drug | Drug Name | Tacrolimus Hydrate | -1.01 | -1.61 | -0.41 | 0.0024095002317386 | ** | 0.0130801441151526 | * |
| JADER | Drug | Drug Name | Dexamethasone | -0.96 | -3.06 | 1.14 | 0.345759366838935 |  | 0.486624294069612 |  |
| JADER | Drug | Drug Name | Valaciclovir Hydrochloride | 2.45 | 1.2 | 3.69 | 0.0005536448777479 | *** | 0.0052596263386056 | ** |
| JADER | Drug | Drug Name | Pregabalin | -1.45 | -3.46 | 0.56 | 0.14286552133443 |  | 0.301604989483797 |  |
| JADER | Drug | Drug Name | Prednisolone | 0.28 | -0.36 | 0.92 | 0.375572876833911 |  | 0.509706047131737 |  |
| JADER | Drug | Drug Name | Lenalidomide Hydrate | -3.46 | -5.39 | -1.52 | 0.0021758284706476 | ** | 0.0130801441151526 | * |
| JADER | Demographics | Age Group | <60 | -1.21 | -3.03 | 0.61 | 0.179695953334357 |  | 0.325164106033597 |  |
| JADER | Demographics | Age Group | ≥60 | 11.31 | 6.88 | 15.75 | 3.31313318813644e-05 | *** | 0.0012589906114918 | ** |
| JADER | Demographics | Age Group | Unknown | 0.79 | -0.18 | 1.76 | 0.104903921082451 |  | 0.284739214366653 |  |
| JADER | Demographics | Gender | F | 5.85 | 3.14 | 8.55 | 0.0002140160299573 | *** | 0.0040663045691889 | ** |
| JADER | Demographics | Gender | M | 3.78 | 0.4 | 7.17 | 0.030302012052516 | * | 0.127941828666179 |  |
| JADER | Demographics | Gender |  | 0.62 | -0.17 | 1.41 | 0.11751511100037 |  | 0.296850253346247 |  |
| JADER | Demographics | Gender | U | -0.37 | -3.45 | 2.71 | 0.779489240158702 |  | 0.822794197945296 |  |
| CVAR | Overall Trend | Overall Trend | Overall | 36.2433653303209 | 30.0303734580134 | 42.4563572026285 | 1.0623361842541e-10 | *** | 1.96532194087009e-09 | *** |
| CVAR | Drug | ATC L1 | N NERVOUS SYSTEM | 26.1874647092044 | 18.127307818686 | 34.2476215997229 | 1.36647044246474e-06 | *** | 6.31992579639942e-06 | *** |
| CVAR | Drug | ATC L1 | L ANTINEOPLASTIC AND IMMUNOMODULATING AGENTS | 93.496329757202 | 62.5919497184522 | 124.400709795952 | 3.68419649554616e-06 | *** | 1.36315270335208e-05 | *** |
| CVAR | Drug | ATC L1 | A ALIMENTARY TRACT AND METABOLISM | 48.2281197063817 | 26.8241675981702 | 69.6320718145931 | 0.000137368916331141 | *** | 0.000211777079343842 | *** |
| CVAR | Drug | ATC L1 | C CARDIOVASCULAR SYSTEM | 12.1225296442691 | 7.22151342783644 | 17.0235458607018 | 4.76777850605031e-05 | *** | 0.000103769296896389 | *** |
| CVAR | Drug | ATC L1 | J ANTIINFECTIVES FOR SYSTEMIC USE | 5.68210050818763 | 3.22017059096992 | 8.14403042540535 | 0.000105442234128729 | *** | 0.00016962446359839 | *** |
| CVAR | Drug | ATC L1 | M MUSCULO-SKELETAL SYSTEM | 2.5578769057031 | 0.899497534797685 | 4.2162562766085 | 0.00432126254389593 | ** | 0.0044412976145597 | ** |
| CVAR | Drug | ATC L1 | G GENITO URINARY SYSTEM AND SEX HORMONES | 5.93788819875791 | 2.97054810124324 | 8.90522829627258 | 0.000468142177536495 | *** | 0.00057737535229501 | *** |
| CVAR | Drug | ATC L1 | D DERMATOLOGICALS | 18.511575381141 | 9.98527011981485 | 27.0378806424672 | 0.000204547971191269 | *** | 0.000302730997363079 | *** |
| CVAR | Drug | ATC L1 | R RESPIRATORY SYSTEM | 13.2015810276683 | 8.03339978905093 | 18.3697622662856 | 3.24618231945653e-05 | *** | 8.57919612999226e-05 | *** |
| CVAR | Drug | ATC L1 | H SYSTEMIC HORMONAL PREPARATIONS, EXCL. SEX HORMONES AND INSULINS | 5.98136645962745 | 4.40460338268946 | 7.55812953656544 | 1.37964193765858e-07 | *** | 7.29239309905247e-07 | *** |
| CVAR | Drug | ATC L2 | N03 ANTIEPILEPTICS | 2.77865612648228 | 1.64594594156449 | 3.91136631140008 | 5.25429126973612e-05 | *** | 0.000108004876100131 | *** |
| CVAR | Drug | ATC L2 | L01 ANTINEOPLASTIC AGENTS | 23.5375494071151 | 18.2393725748507 | 28.8357262393794 | 1.1199591567717e-08 | *** | 6.90641480009214e-08 | *** |
| CVAR | Drug | ATC L2 | N02 ANALGESICS | 14.0118577075102 | 9.25596242794491 | 18.7677529870755 | 5.26818742540777e-06 | *** | 1.77202667945534e-05 | *** |
| CVAR | Drug | ATC L2 | N05 PSYCHOLEPTICS | 5.51383399209495 | 3.76949259010228 | 7.25817539408763 | 2.0120508309196e-06 | *** | 8.27176452711393e-06 | *** |
| CVAR | Drug | ATC L2 | N06 PSYCHOANALEPTICS | 3.09994353472624 | 1.46048275823704 | 4.73940431121544 | 0.000801542500884231 | *** | 0.000926783516647393 | *** |
| CVAR | Drug | ATC L2 | J01 ANTIBACTERIALS FOR SYSTEMIC USE | 4.9870129870131 | 2.54446227411044 | 7.42956369991576 | 0.000383976160629593 | *** | 0.000507397069403391 | *** |
| CVAR | Drug | ATC L2 | L04 IMMUNOSUPPRESSANTS | 69.2111801242249 | 43.1548794092419 | 95.2674808392078 | 2.00931544976243e-05 | *** | 6.19538930343416e-05 | *** |
| CVAR | Drug | ATC L2 | A02 DRUGS FOR ACID RELATED DISORDERS | 7.58215697346152 | 3.44730586568756 | 11.7170080812355 | 0.00105872811933933 | ** | 0.00118705880047137 | ** |
| CVAR | Drug | ATC L2 | A01 STOMATOLOGICAL PREPARATIONS | 13.0389610389613 | 7.51735872649503 | 18.5605633514277 | 8.15122223245178e-05 | *** | 0.000146405175474007 | *** |
| CVAR | Drug | ATC L2 | D11 OTHER DERMATOLOGICAL PREPARATIONS | 5.46527385657818 | 3.12956029237369 | 7.80098742078267 | 9.04245971117492e-05 | *** | 0.000152077731506124 | *** |
| CVAR | Drug | Drug Name | ABATACEPT | 8.33333333333359 | 4.64575973125864 | 12.0209069354085 | 0.000226337444153159 | *** | 0.000310166127172847 | *** |
| CVAR | Drug | Drug Name | ACETAMINOPHEN | 6.07905138339934 | 3.25960841622822 | 8.89849435057047 | 0.000220006539330065 | *** | 0.000310166127172847 | *** |
| CVAR | Drug | Drug Name | ADALIMUMAB | 6.48406374501973 | 3.96015318192011 | 9.00797430811935 | 3.96083419588345e-05 | *** | 9.77005768317917e-05 | *** |
| CVAR | Drug | Drug Name | ETANERCEPT | 5.9882828846424 | 3.0037268210971 | 8.9728389481877 | 0.000560062381566988 | *** | 0.000668461552192857 | *** |
| CVAR | Drug | Drug Name | INFLIXIMAB | 5.9073969508753 | 3.47315616906837 | 8.34163773268222 | 5.95834933234037e-05 | *** | 0.000116031013313997 | *** |
| CVAR | Drug | Drug Name | METHOTREXATE | 7.55004721435309 | 4.48780077237488 | 10.6122936563313 | 8.30948293230852e-05 | *** | 0.000146405175474007 | *** |
| CVAR | Drug | Drug Name | OXYCODONE | 5.63335340156546 | 2.87390206113181 | 8.39280474199911 | 0.000411029049986775 | *** | 0.000524416374121058 | *** |
| CVAR | Drug | Drug Name | PREDNISONE | 5.68562046318704 | 3.41625960437346 | 7.95498132200062 | 4.62507331667447e-05 | *** | 0.000103769296896389 | *** |
| CVAR | Drug | Drug Name | RITUXIMAB | 6.66606468560185 | 4.2139587664148 | 9.11817060478889 | 2.42574512112084e-05 | *** | 6.904043806267e-05 | *** |
| CVAR | Drug | Drug Name | TOCILIZUMAB | 11.415482423336 | 5.1984371353886 | 17.6325277112834 | 0.00194397342156084 | ** | 0.0020550576170786 | ** |
| CVAR | Demographics | Age Group | <60 | 12.2755505364203 | 9.59322460034387 | 14.9578764724968 | 6.86659467104649e-09 | *** | 5.0812800565744e-08 | *** |
| CVAR | Demographics | Age Group | >=60 | 20.7425183512143 | 17.5577637173687 | 23.92727299 | 1.47194840895396e-11 | *** | 5.44620911312966e-10 | *** |
| CVAR | Demographics | Age Group | unknown | 3.22529644268786 | 1.40922624869965 | 5.04136663667606 | 0.00140205087545254 | ** | 0.001525761246816 | ** |
| CVAR | Demographics | Gender | Male | 13.6476566911349 | 10.9901168934526 | 16.3051964888172 | 9.82582557714912e-10 | *** | 9.08888865886294e-09 | *** |
| CVAR | Demographics | Gender | Female | 22.1089779785436 | 18.0709337312003 | 26.1470222258869 | 3.24100969434534e-10 | *** | 3.99724528969258e-09 | *** |
| CVAR | Demographics | Gender |  | 0.486730660643724 | -0.055686836 | 1.02914815689202 | 0.0759319675585521 |  | 0.0759319675585521 |  |
